# Identification of Nucleotide-Binding Sites in Protein Structures: A Novel Approach Based on Nucleotide Modularity

**DOI:** 10.1371/journal.pone.0050240

**Published:** 2012-11-27

**Authors:** Luca Parca, Pier Federico Gherardini, Mauro Truglio, Iolanda Mangone, Fabrizio Ferrè, Manuela Helmer-Citterich, Gabriele Ausiello

**Affiliations:** Department of Biology, University of Rome “Tor Vergata”, Rome, Italy; Universität Erlangen-Nürnberg, Germany

## Abstract

Nucleotides are involved in several cellular processes, ranging from the transmission of genetic information, to energy transfer and storage. Both sequence and structure based methods have been developed to predict the location of nucleotide-binding sites in proteins. Here we propose a novel methodology that leverages the observation that nucleotide-binding sites have a modular structure. Nucleotides are composed of identifiable fragments, i.e. the phosphate, the nucleobase and the carbohydrate moieties. These fragments are bound by specific structural motifs that recur in proteins of different fold. Moreover these motifs behave as modules and are found in different combinations across fold space. Our method predicts binding sites for each nucleotide fragment by comparing a query protein with a database of templates extracted from proteins of known structure. Whenever a similarity is found the fragment bound by the template is transferred on the query protein, thus identifying a putative binding site. Predictions falling inside the surface of the protein are discarded, and the remaining ones are scored using clustering and conservation. The method is able to rank as first a correct prediction in the 48%, 48% and 68% of the analyzed proteins for the nucleobase, carbohydrate and phosphate respectively, while considering the first five predictions the performances change to 71%, 65% and 86% respectively. Furthermore we attempted to reconstruct the full structure of the binding site, starting from the predicted positions of the fragments. We calculated that in the 59% of the analyzed proteins the method ranks as first a reconstructed binding site or a part of it. Finally we tested the reliability of our method in a real world case in which it has to predict nucleotide-binding sites in unbound proteins. We analyzed proteins whose structure has been solved with and without the nucleotide and observed only little variations in the method performance.

## Introduction

Nucleotides are ubiquitous molecules in the cellular environment and they are involved in key cellular processes. They store and transfer energy and serve as building block of nucleic acids and enzyme cofactors. Nucleotides were also one of the earliest cofactors to be bound by proteins [1]. Indeed nucleotide-binding folds, such as the Rossmann-type [2] and the P-loop containing nucleotide hydrolases folds [3], are ancient and widespread.

Several nucleotide-binding site prediction methods have been developed, relying both on sequence and structural information. Sequence-based methods use machine learning techniques to identify nucleotide-binding residues based on characteristics such as conservation or structural features of residues, like hydrophobicity, solvent accessibility or net charge.

Chauhan et al. developed methods for the identification of ATP [4] and GTP [5] binding residues with accuracies of 66% and 68% respectively, while Ansari et al. designed a method specifically for NAD [6] reaching an accuracy of 74%. However these methods do not give any insights into the conformation of the bound nucleotide and the details of its interaction with the protein residues.

From the structural point of view several studies have investigated the structural features that a protein must possess in order to bind a nucleotide. As mentioned some folds are specifically associated to the binding of nucleotides. Besides this coarse observation, efforts [7]–[15] have been made to discover which local features, such as structural motifs or a specific physicochemical environment, are required to bind a nucleotide, or a specific fragment thereof. The results of these studies show that some chemical fragments, like the phosphate or the nucleobase moiety, are bound by specific structural motifs which are shared by non-homologous proteins having a different fold. Moreover the motifs that bind these different moieties behave as modules and are reused in different combinations across fold-space [16]. This observation suggests the possibility of predicting nucleotide-binding sites starting form the identification of structural motifs for their constituent fragments.

**Table 1:** Minimum distance allowed for nucleotide modules from the protein surface.

Nucleotide modules	Minimum distance from the surface (Å)
Nucleobase	0.407
Carbohydrate	0.124
Phosphate	0.830

Minimum distance, in Ångström, allowed for a predicted nucleotide module from the solvent excluded surface. The distance is calculated considering any atom of the nucleotide module and any vertex of the mesh representing the solvent excluded surface.

For instance Saito et al [17] developed a method that identifies nucleotide-binding site positions using an empirical scoring system derived from the spatial distributions of protein atoms around the nucleobase moiety. The performance of this method reaches values ranging from 30% and 40% of analyzed protein structures in which the first ranked prediction is correct. Moreover another method [18] has been developed that uses known chemical fragment-fragment interactions with the aim of building, with a docking-like methodology, energetically favoured nucleotide conformations on a protein surface. In 53% of the analyzed proteins this method placed a correct nucleotide conformation in the first rank. We recently developed a method for the identification of phosphate binding sites, based on the identification of specific structural motifs [19], [20]. Here we extend this approach to the other nucleotide fragments. Our method searches for similarities between a query protein structure and a reference set of template binding sites for nucleotide modules. Whenever a similarity is found the module bound by the template binding site is transferred on the query protein identifying a putative module-binding site. Furthermore we explore the possibility of using the predicted positions of nucleotide fragments to reconstruct the structure of the full nucleotide. Here we demonstrate that this method is reliable when analyzing protein structures without a bound nucleotide (the real world case) and show that has a better performance than a previously developed method. The method is available and downloadable at http://pdbfun.uniroma2.it/nucleos.

**Table 2:** Minimum and maximum distances allowed between nucleotide modules.

Pair of nucleotide modules	Minimum distance (Å)	Maximum distance (Å)
Nucleobase–Carbohydrate	3.937	5.275
Phosphate–Carbohydrate	3.079	5.151
Nucleobase–Phosphate	5.189	19.063
Nucleobase–Nucleobase	8.846	19.540
Carbohydrate–Carbohydrate	5.528	16.333
Phosphate–Phosphate	2.654	7.148

These distances (in Ångström) are calculated between the centroids of nucleotide modules.

## Materials and Methods

### Nucleotide Modules Binding Sites Datasets

We define nucleotides as composed of nucleobases, carbohydrates and phosphates. The nucleobase module can be adenine, guanine, cytosine, thymine, uracil and the nicotinamide and flavin moieties from respectively the NAD and FAD molecules. The carbohydrate can be the ribose in closed and open form and the deoxyribose ring. We collected binding sites for these modules from all the structures in the Protein Data Bank (PDB) [21], March 2012 version. Whenever a ligand (excluding nucleic acid molecules) bound by a protein structure contains one of these modules we collect all the residues that have at least one non-hydrogen atom in a radius of 3.5 Å from any non-hydrogen atom of the nucleotide module. Since the structural comparison method [22] used in this work requires input structures of at least three residues, binding sites composed of less than three residues were discarded. In order to reduce redundancy we used BLASTClust [23] with a 95% sequence identity threshold. A single binding site for each type of module was randomly selected from each cluster of sequences defined by BLASTClust.

We collected 4657 binding sites for nucleobases, 3073 for carbohydrates and 10185 for phosphates.

**Table 3:** F-score of the method at different R.M.S.D. thresholds.

R.M.S.D. threshold (Å)	Nucleobase	Carbohydrate	Phosphate
0.6	0.48	0.47	0.64
0.7	0.43	0.42	0.50
0.8	0.38	0.34	0.42

Average F-scores of the method (considering all the nucleotide types) for each nucleotide module and for each R.M.S.D. threshold used during the structural comparison step.

### Protein Structure Datasets

Two datasets of protein structures were used to test the method in this work. The first dataset is composed of 1925 protein-nucleotide complexes from the sc-PDB [24] (this dataset will be referred from now on as sc-PDB dataset) database. Different types of nucleotides are represented in this dataset: adenosine mono- di- and tri-phosphate (respectively AMP, ADP and ATP), guanosine di- and tri-phosphate (GDP and GTP), phosphoaminophosphonic acid-adenylate ester (ANP), phosphoaminophosphonic acid-guanylate ester (GNP), nicotine-adenine dinucleotide (NAD), nicotinamide-adenine-dinucleotide phosphate(NAP), flavine-adenine dinucleotide (FAD), flavin mononucleotide (FMN). For each of these proteins we selected the chain that binds the nucleotide with the highest number of residues. A residue is defined as contacting the nucleotide if it has at least one non-hydrogen atom in a 3.5 Å radius from any non-hydrogen atom of the nucleotide. This step discarded weakly bound ligands and nucleotides bound at the interface of multiple protein chains. In order to remove homologous protein chains we used BLASTClust with a 30% sequence identity threshold. For each non-redundant group of proteins binding a given nucleotide we selected, as representative, the structure with the best resolution. After removing redundancy the dataset contains 1039 nucleotide-binding proteins. The surface cannot be calculated if the structure contains artifacts or missing coordinates, while the residue conservation score requires the protein to be present in PFAM and a good correspondence between the Uniprot and PDB sequences. These requirements lead to the removal of 115 structures reducing the size of the dataset to 924 nucleotide-binding protein structures (the complete list is provided in Table S1).

The second dataset is composed of proteins from the LigASite [25] database that bind nucleotides and that have been crystallized in both their apo and holo forms. The LigASite database contains 391 non-redundant pairs of proteins (January 2012 release). The apo and holo structures of the same protein must share the same nucleotide-binding chains. For each protein we considered only one nucleotide-binding chain that must be common to both apo and holo structures of the protein. As before we selected the chain with the highest number of nucleotide-binding residues. 72 of the 391 proteins bind one of the eleven nucleotide types considered in this work and their apo and holo structures share the nucleotide-binding chain. The calculation of solvent excluded surface of the protein and of the residue conservation was not possible for 8 proteins because of the aforementioned issues. The final number of pairs of apo-holo structures is then reduced to 64 (the complete list is provided in Table S2).

**Table 4:** Results of the method considering the nucleotide type.

	Nucleobase			Carbohydrate			Phosphate		
Ligand	Precision	Recall	F-score	Precision	Recall	F-score	Precision	Recall	F-score
AMP	0.36	0.26	0.29	0.52	0.31	0.38	0.29	0.43	0.32
ADP	0.41	0.36	0.37	0.32	0.34	0.32	0.82	0.76	0.78
ATP	0.51	0.31	0.38	0.44	0.42	0.43	0.71	0.68	0.69
ANP	0.44	0.43	0.42	0.34	0.38	0.25	0.86	0.69	0.74
FAD	0.51	0.41	0.43	0.75	0.52	0.6	0.82	0.65	0.71
FMN	0.21	0.69	0.32	0.27	0.38	0.21	0.64	0.42	0.47
GDP	0.94	0.59	0.71	0.56	0.52	0.52	0.86	0.80	0.81
GTP	0.45	0.28	0.27	0.23	0.36	0.05	0.78	0.53	0.61
GNP	0.75	0.75	0.78	0.43	0.65	0.56	0.98	0.90	0.92
NAD	0.41	0.56	0.47	0.72	0.55	0.61	0.54	0.39	0.45
NAP	0.7	0.41	0.5	0.8	0.49	0.59	0.56	0.47	0.49

Complete results reporting precision, recall and F-score for all the nucleotide types and for each nucleotide module considered. These results have been obtained using 0.6 Å as R.M.S.D. threshold.

### Method Overview

The method is divided in three steps: i) identification of binding sites for the three types of nucleotide modules: nucleobase, carbohydrate and phosphate, ii) filtering and clustering of the predicted modules, iii) scoring and ranking of the predicted binding sites.

#### Identification of binding sites for nucleobases, carbohydrates and phosphates

The method uses the Superpose3D local structural comparison software [22] based on the Query3D algorithm [26], to identify structural similarities between a query protein structure and a set of template binding sites for one of the three types of nucleotide module. Briefly, Superpose3D searches for the largest subset of amino acids that have a similar conformation in two protein structures. Proteins are represented as an ensemble of non-connected residues, so that sequence collinearity is not required, and each amino acid is represented by its Cα and the geometric center of its side chain. Each structural match is evaluated by three criteria: i) the Root Mean Square Deviation (R.M.S.D.) must be equal or lower than a specified threshold, ii) matching amino acids must have a BLOSUM62 [27] substitution score equal or greater than a specified threshold, iii) matching amino acids must be close to at least one amino acid in the set (distance between their Cα equal or lower than 7.5 Å).

Whenever Superpose3D finds a structural similarity between a set of amino acids from the query protein structure and a binding site for a nucleotide module, this nucleotide module is rotated and translated onto the query protein structure using the same 3D transformation associated with the structural match. Thus the nucleotide module is added to the analyzed protein and represents a putative binding site for that nucleotide module.

**Figure 1:**
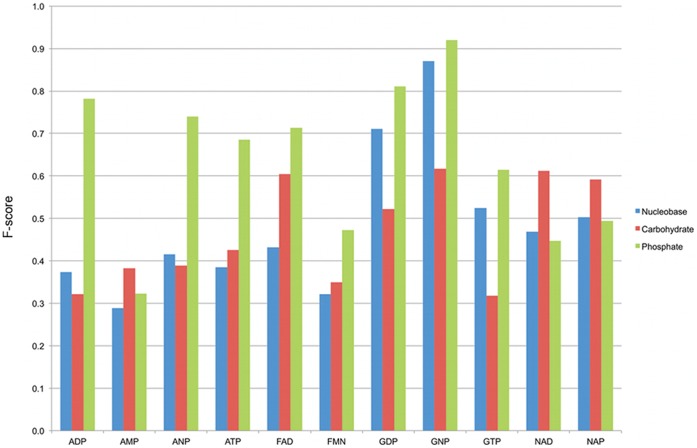
F-scores measuring the method performance for the different nucleotides. Complete results reporting the F-scores for all the nucleotide types and for each nucleotide module considered. The bars are colored depending on the nucleotide module: blue for the nucleobase, red for the carbohydrate and green for the phosphate.

#### Filtering and clustering of the predicted nucleotide modules

The principal aim of this method is to identify binding sites for nucleotides on newly solved protein structures where homology-based binding site identification is not possible. We simulate this situation and test the ability of the method in finding nucleotide-binding sites in a protein structure without using template binding sites from homologous protein structures. To this end all the predictions derived from structural matches involving potentially homologous proteins are discarded. For each structural match we aligned the query protein chain and the chain whose template binding site is involved in the structural match with the Needle alignment program from the Emboss suite [28]. If the sequence identity between the two protein chains is equal or higher than 30% the prediction is discarded.

Predicted nucleotide modules are discarded if they have at least one atom inside the surface of the protein. The Solvent Excluded Surface [29] is calculated using the UCSF Chimera MSMS package [30]. Nevertheless the remaining predicted modules could be too close to the protein surface to represent real ligand positions. Therefore we analyzed the minimum distance between each type of nucleotide module and the protein surface in a set of non-redundant nucleotide-binding protein structures, excluding those that are potentially homologous to proteins included in our test sets. The method accepts only those predictions whose distance from the protein surface is not smaller than the minimum distance for the corresponding module (Table 1) in this dataset. Finally predicted modules of the same type are clustered together using an agglomerative hierarchical clustering procedure, in a centroid linkage fashion, with a threshold of 2 Å. For each cluster the method selects the module closest to the centroid of the cluster.

**Figure 2:**
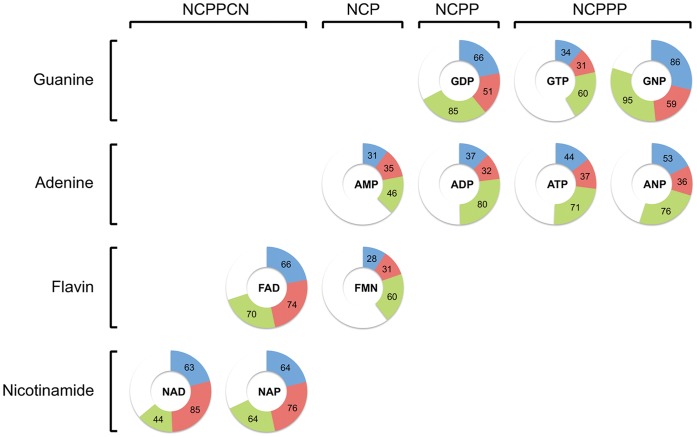
Performance of the method considering first-ranked predictions. Complete results, as percentage of analyzed protein structures in which the method places a correct prediction in the first rank for the three types of nucleotide modules. Each nucleotide type is represented by a doughnut chart that is divided in three sectors, one for each nucleotide module (blue for the nucleobase, red for the carbohydrate and green for the phosphate). The size of each module sector is proportional to the percentage of proteins in which the method is successful. The columns divide the nucleotides by their architecture type: NCP stands for nucleobase-carbohydrate-phosphate (e.g. the AMP) and so on. Moreover nucleotides are grouped by their characteristic molecular feature so that rows divide them in guanine-, adenine-, flavin- and nicotinamide-containing nucleotides.

#### Scoring of the predicted nucleotide-binding sites

Each prediction on the protein surface is assigned a score. The score for a predicted binding site for a nucleotide module is composed of two parts: i) a clustering score, equal to the number of the modules of the same type that clustered with it, ii) a conservation score, that is the average conservation of the query protein residues involved in its structural match.

This conservation score is calculated for each residue in the protein structure, as described in a previous work [19], taking into account the PFAM [31] multiple alignment of the protein family. Briefly, for each residue the percentage of similar residues (BLOSUM62 matrix score equal or higher than 1) in its PFAM alignment column is calculated. These percentages are normalized to percentiles that represent the conservation scores, using the distribution of percentage values for that multiple alignment. This procedure normalizes the conservation scores from different PFAM multiple alignments and makes them comparable.

Each predicted nucleotide-binding site is given a score that is the sum of the clustering scores of its nucleotide modules plus their conservation score.

**Table 5:** Overall results of the method after ranking predictions.

Top ranks	Nucleobase	Carbohydrate	Phosphate
1	48%	48%	68%
3	66%	62%	82%
5	71%	65%	86%
10	76%	67%	90%

Complete results, as percentages of analyzed protein structures in which the method places a correct prediction of the three types of nucleotide modules considering the 1, 3, 5 and 10 top ranks.

### Combination of Binding Sites for Nucleotide Modules

In order to reconstruct nucleotide-binding sites from their predicted modules we derived a set of rules, in the form of distance constraints that are used when joining the modules to build nucleotide-binding sites. We analyzed the distances between all the possible pairs of nucleotide modules in a set of 1226 non-redundant (30% sequence identity threshold) set of nucleotide-binding proteins culled from the PDB. Protein chains showing a sequence identity equal or higher than 30% with any of the protein structures analyzed by the method were discarded. For each pair of nucleotide modules we analyzed the distribution of centroid distances and defined the minimum and maximum allowed distances in a way that discarded 1% of the lowest and 1% of the highest distances. All these distances are reported in the Table 2.

These distance thresholds are used when joining modules to reconstruct the full nucleotide. The aim is to build combination of modules that follow the architecture of the nucleotide bound by the protein, e.g. the FAD molecule has a nucleobase-carbohydrate-phosphate-phosphate-carbohydrate-nucleobase architecture. If the predicted modules do not permit the full reconstruction of the nucleotide, the method tries, iteratively, to build a “sub-architecture” of the bound nucleotide, e.g. the ADP molecule is a sub-architecture of the ATP molecule. The minimum allowed sub-architecture is composed of two consecutive nucleotide modules, e.g. nucleobase-carbohydrate or carbohydrate-phosphate.

**Table 6:** Results of the method after ranking predictions considering the nucleotide type.

	Nucleobase				Carbohydrate				Phosphate			
Ligand	Best	Top 3	Top 5	Top 10	Best	Top 3	Top 5	Top 10	Best	Top 3	Top 5	Top 10
AMP	31	55	58	61	35	49	52	52	46	64	75	86
ADP	37	55	60	66	32	48	51	53	80	92	94	97
ATP	44	63	66	73	37	53	56	58	71	85	88	96
ANP	53	63	71	74	36	51	58	63	76	90	91	99
FAD	66	84	87	92	74	85	86	88	70	85	86	99
FMN	28	65	74	82	31	51	59	60	60	76	81	95
GDP	66	70	70	77	51	72	77	77	85	89	91	98
GTP	34	46	46	54	31	51	57	57	60	71	74	96
GNP	86	91	95	95	59	77	82	86	95	100	100	100
NAD	63	83	88	89	85	88	89	89	44	68	74	92
NAP	64	76	78	83	76	80	85	88	64	78	86	90

Complete results for the sc-PDB dataset divided by nucleotide type, as percentage of analyzed protein structures in which the method places a correct prediction in the first, top three, top five and top ten predictions for the three types of nucleotide modules.

## Results

### Overview

We developed a method for the identification of binding sites for nucleotide molecules on protein structures. This method compares the structure of a query protein with three datasets of template binding sites, one for each type of nucleotide module: the nucleobase, the carbohydrate and the phosphate. Predicted nucleotide modules are used to identify putative binding sites for the nucleotide bound by the analyzed proteins. The method has been tested on two datasets of protein structures. The first dataset contains protein structures binding several common nucleotides with different architectures, the second datasets contains nucleotide-binding protein structures that have been solved in both apo and holo forms. After predicting binding sites for nucleotide modules on a protein structure, the method ranks the predictions according to their score. A prediction for a specific module is considered correct if its centroid lies at 5 Å or less from the crystallographic position.

**Figure 3:**
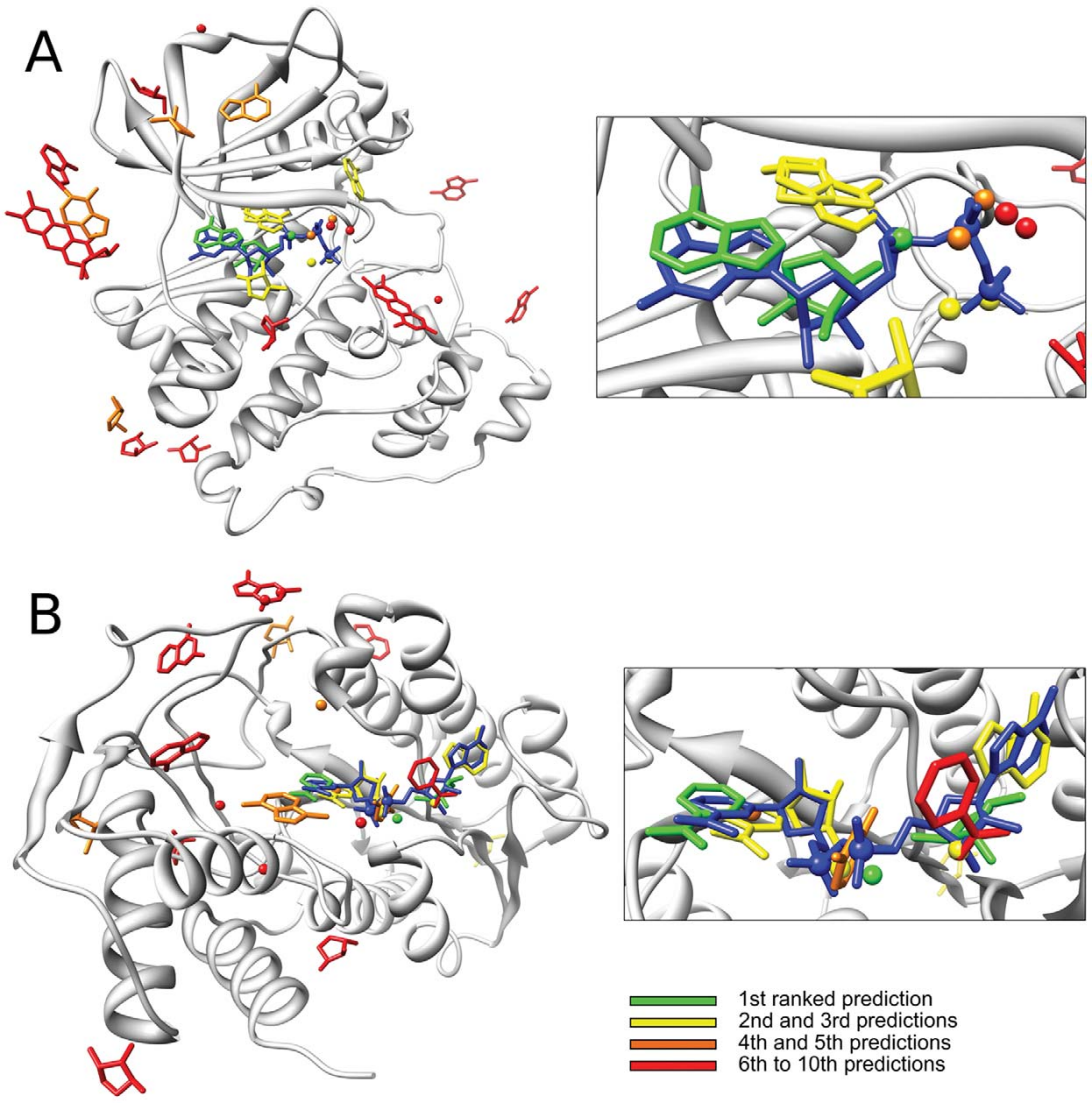
Two test cases of the ranking system. Two protein structures with ranked predictions (only the top ten for each type of nucleotide module are shown). The first ranked prediction is colored in green, the 2^nd^ and the 3^rd^ are colored in yellow, the 4^th^ and the 5^th^ are colored in orange, from the 6^th^ to the 10^th^ predictions are colored in red. The nucleotides complexed with proteins are colored in blue. A: structure of the kinase CK2 from *Z. mais* (PDB: 1day) complexed with phosphoaminophosphonic acid-guanylate ester (GNP). B: structure of the DTDP-glucose oxidoreductase from *S. enterica* (PDB: 1n2s) complexed with NAD.

**Table 7:** Comparison of the method performance on apo and holo structures.

	Nucleobase				Carbohydrate				Phosphate			
Structure form	1	3	5	10	1	3	5	10	1	3	5	10
Holo	48	66	67	70	33	50	55	58	53	72	73	80
Apo	38	62	68	71	40	49	52	58	49	66	71	82

Complete results for the LigASite dataset (apo-holo structures), as percentage of analyzed protein structures in which the method places a correct prediction in the first, top three, top five and top ten predictions for the three types of nucleotide modules.

### Results on the sc-PDB Dataset

The first analyzed dataset is composed of 924 protein structures binding AMP, ADP, ATP, GDP, GTP, ANP, GNP, FAD, FMN, NAD and NAP.

We first searched for an optimal combination of parameters to use in order to screen the predictions, evaluating the performance with a ten-fold cross validation test. For each test we:

- divide a group of proteins binding the same nucleotide, say ATP, into training and test sets with the sizes of the sets respecting a 9∶1 ratio;
- analyze the predictions made on the training set proteins searching for the scoring threshold that gives the best F-score (harmonic mean of precision and recall) for the method performance;
- evaluate the performance, using the F-score, that the method achieves on the test set when using the threshold selected in the previous step.

The performance of the method is evaluated by calculating precision, recall and F-score. For each nucleotide, and for each type of nucleotide module, the average F-score achieved in the ten tests is calculated. The whole procedure has been conducted with different R.M.S.D. thresholds in the structural comparison step: 0.6, 0.7 and 0.8 Å. A small variation of the R.M.S.D. threshold influences dramatically the number of predictions made on a protein. The results show that the best R.M.S.D. is 0.6 Å since it has the best average F-scores throughout all the nucleotide types: 0.48, 0.47 and 0.64 on nucleobases, carbohydrates and phosphates respectively. Complete results are reported in Table 3. From now on all the results reported use 0.6 Å as R.M.S.D threshold. These results show that higher R.M.S.D. thresholds result in a greater number of structural matches thus improving the recall of the method (the amount of correctly identified binding sites). However the F-score decreases dramatically because of the amount of false positive predictions, thus decreasing the precision of the method (complete results divided by nucleotide type are reported in Table 4). It is also clear that the phosphate module is the one that better identifies binding sites for nucleotides compared to carbohydrate and nucleobase.

**Figure 4:**
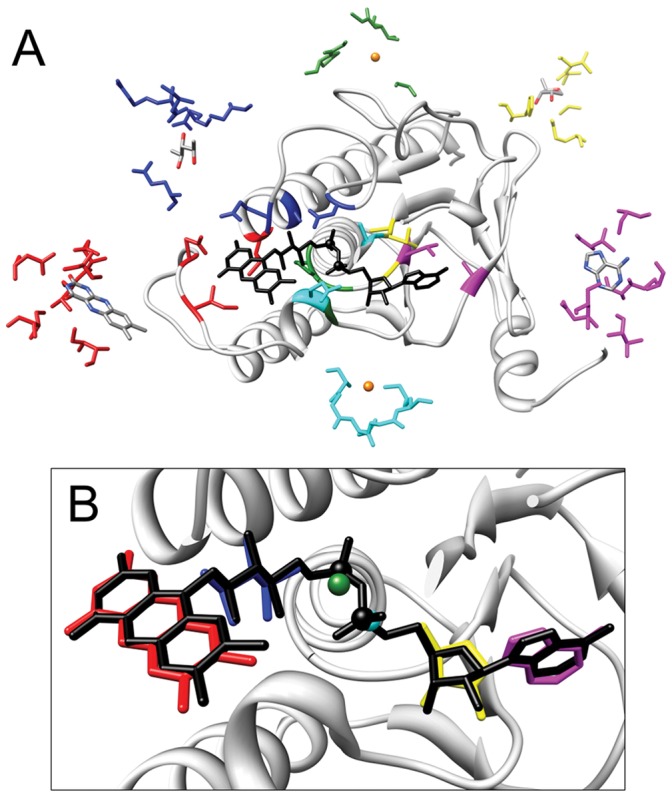
Test case for linked predicted binding sites forming a FAD-binding site. Top ranked prediction for the binding site of the FAD molecule bound by the subunit F of the Alkyl hydroperoxide reductase from *S. typhymurium* (PDB code 1hyu). A) Structure of the reductase (residues from 1 and 197 and from 325 to 453 are not shown for convenience) with the structures of the template binding sites matching with reductase residues (red: I46, N48, V290, Q289 of the thioredoxin reductase from *H. pylori* (PDB: 3ish) binding a flavin module; blue: D287, Q295, S299 of the thioredoxin reductase from *S. cerevisiae* (PDB: 3itj) binding a ribose; cyan: K112, G109, S108 of the human RNA helicase DDX20 (PDB: 3b7g) binding a phosphate; green: G10, P11, G38, G138 of the 2-oxoglutarate dehydrogenase E3 component from *T. thermophilus* (PDB: 2yqu) binding a phosphate; yellow: G10, A137, T138 of a putative monoxygenase from *S. aureus* (PDB: 3d1c) binding a ribose; magenta: I11, A122, T157 of the gernaylgeranyl reductase from *S. acidocaldarius* (PDB: 3atr) binding an adenine). The modules bound by the template binding sites are colored by atom type (grey for Carbon, blue for Nitrogen, Red for Oxygen and Orange for Phosphorus). Matching residues are depicted with the same color. B) The prediction is composed of predicted binding sites for nucleotide modules: predicted modules are colored as their binding sites in A. The FAD molecule bound by the protein is colored in black.

With some nucleotide types the method is more successful than with others: GNP for example has a performance of 0.87, 0.62 and 0.92 for the nucleobase, the carbohydrate and the phosphate respectively (results will be written in this order from now on). In the majority of cases the binding site for a particular nucleotide cannot be identified with all the nucleotide modules: the performance of the method on FAD-binding proteins is 0.43, 0.60 and 0.71, while on the ATP-binding proteins it has a performance of 0.38,0.43 and 0.69. Complete results are in Figure 1.

Moreover we also ranked the predictions irrespective of any scoring threshold and evaluated the performance according to the position of the correct predictions in the ranked list. Therefore, for each module, we calculated the percentage of proteins for which the method places a correct prediction among the top 1, 3, 5 and 10. Before ranking the predictions we observe that in 77%, 67% and 93% of the protein structures there is at least one correct prediction for the nucleobase, the carbohydrate and the phosphate respectively. A correct prediction is ranked first in 48%, 48% and 68% of the analyzed proteins for the nucleobase, carbohydrate and phosphate respectively. Considering the top five predictions the method reaches a performance of 71%, 65% and 86% (Table 5). The performance of the method varies according to the identity of the nucleotide. Considering the first ranked prediction the method reaches a performance of 66%, 74% and 70% on FAD binding proteins and 86%, 59% and 95% on GNP binding proteins. On the other hand worse performances are obtained with smaller nucleotides such as AMP and FMN (31%, 35% and 46% on AMP binding proteins and 28%, 31% and 60% on FMN binding proteins). The results obtained for each nucleotide type when considering the first ranked prediction are graphed in Figure 2 (complete results, divided by nucleotide type, considering also the top three, five and ten prediction are reported in Table 6).

Overall the method performs better on bigger nucleotides such as FAD, NAD and NAP than on smaller ones such as FMN and AMP. Moreover the phosphate module is the one that better identifies nucleotide-binding sites, while the carbohydrate and nucleobase modules identify the correct binding sites depending on the type of the nucleotide. Figure 2 shows that the nucleobase works better with big nucleotides such as FAD, NAD and NAP and on nucleotides with guanine like GDP and GNP, while the carbohydrate works better than the others on FAD, NAD and NAP. Two cases with the ranked predictions are reported in Figure 3, where the ranking system places correct predictions in the first rank (colored in green). Globally the predicted binding sites rank better as they become close to the real binding site for the nucleotide, showing that scoring system is working as expected. More specifically the method is able to put in the first ranks multiple correct predictions. Figure 3-B shows how the method puts in the first and second ranks a correct prediction respectively for the nicotinamide and for the adenine modules of the NAD molecule complexed with the DTDP-glucose oxidoreductase from *S. enterica* (PDB: 1n2s).

When searching for structural similarities between the query protein and the template binding sites, the method discards matches involving potentially homologous proteins. Similarly it is interesting to quantify how many times a query nucleotide-binding site is identified by a template binding site having a different protein fold. To this end we analyzed the 410 (out of 924) proteins in the dataset that are classified in the SCOP database [32]. Moreover we could not consider predictions made by template binding sites that do not have a SCOP record. The top ranked, correct, prediction has a SCOP fold different from the query protein in 50% and 52% of cases for the nucleobase and carbohydrate respectively. This percentage rises to 86% for phosphate. These results confirm that similar binding motifs for these modules occur in different protein folds [16], [19].

**Figure 5:**
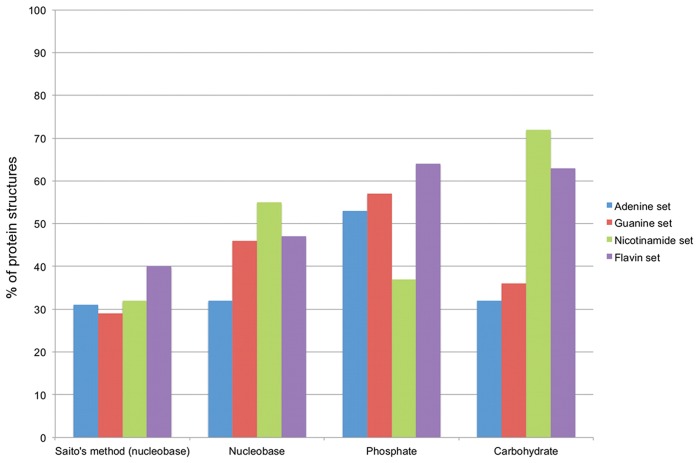
Comparison with another method for the prediction if nucleotide-bingind sites. Comparison between the method developed by Saito [17] and the methodology presented in this work. The first group of bars on the left represents the performance of Saito’s method. The other groups of bars represent the performance of our method for the different nucleotide modules. Bars are colored depending on the protein dataset: proteins binding adenine- (blue), guanine- (red) nicotinamide- (green) and flavin-containing (purple) nucleotides.

**Figure 6:**
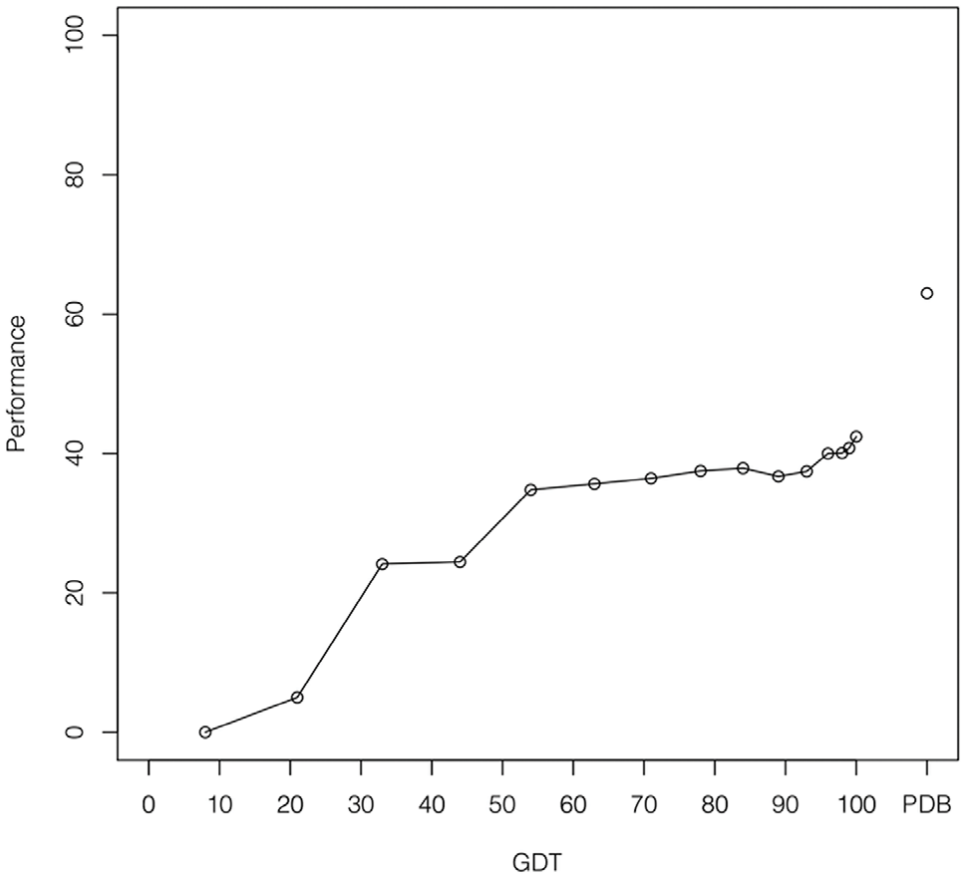
Performance of the method on protein homology models. The performance of the method (Y axis), measured as the percentage of protein structures in which the first-ranked prediction correctly identifies the true nucleotide-binding site, plotted as a function of the GDT value (X axis) of the homology-models.

### Results on Apo-holo Protein Structures

Since the aim of this method is to predict nucleotide-binding sites on unbound structures, we assessed how the performance of the method is affected by the conformational changes that occur upon ligand binding. We used LigASite to compile a dataset of 64 proteins whose structures have been solved both in the bound and unbound state (holo and apo forms respectively). We predicted binding sites for nucleobases, carbohydrates and phosphates on the holo and apo forms of the dataset proteins. When considering the first ranked prediction the method puts a correct prediction in the first rank respectively in the 48%, 33% and 53% of the holo structures, while on the corresponding apo structures these percentages change respectively to 38%, 40% and 49%. Therefore we observed on average a 7% difference between the performances on the apo and holo structures; moreover the carbohydrate has a better performance on apo structures than on holo structures. Considering the top five ranks the method identifies a correct binding site in the 67%, 55% and 73% on holo structures and 68%, 52% and 71% on apo structures showing an average performance gap of only 2% (Complete results are reported in Table 7). This small difference allows the method to be reliable when predicting a nucleotide-binding site on a protein structure crystallized without the ligand.

### Linking Predicted Binding Sites for Nucleotide Modules

We decided to investigate whether binding sites for different nucleotide modules can be predicted independently and subsequently combined together in order to predict the position of the full nucleotide. We derived a set of “centroid distance rules” to use when combining predicted modules. These rules were derived from a non-redundant set of nucleotide-binding proteins and consist of maximum and minimum allowed distances between every pair of nucleotide modules (Table 2). The aim is to build whole nucleotide-binding sites on the surface of the analyzed proteins using the predicted nucleotide modules by joining them in different combinations. The method tries to reconstruct the same architecture of the nucleotide bound by the protein starting from the predicted positions of the individual modules. If a full reconstruction is not possible, the method tries to build “sub-architectures” (the ADP molecule is a “sub-architecture” of the ATP molecule). The minimum allowed sub-architecture consists of two consecutive different nucleotide modules, i.e. nucleobase-carbohydrate or carbohydrate-phosphate. A prediction is scored by summing the scores of its constituent modules.

We evaluated the method using three different criteria to consider a predicted nucleotide-binding site as correct: i) all the modules composing the predicted binding site lie at 5 Å or less from the corresponding modules of the crystallized ligand (“all-modules distance”); ii) the cluster of modules composing the predicted nucleotide-binding site and the cluster of the corresponding nucleotide modules in the crystallized ligand have their centroid laying at less than 5 Å (“ligand-centroid distance”); iii) the RMSD of the modules in the predicted binding sites with the corresponding modules in the crystallized ligand is equal to or lower than 5 Å. After combining nucleotide modules on the 924 protein structures of the sc-PDB dataset the method places a correct prediction in the first rank in 43%, 56% and 59% of the cases using respectively the all-modules distance, the ligand-centroid distance and the RMSD criterion. We observed that, if we consider all the predictions irrespective of their rank, in 63% of the analyzed structures there is at least one correct prediction, using the ligand-centroid distance criterion.

In 98% of the cases all the modules are predicted from templates derived from protein chains having sharing less than 30% sequence identity between them. This result shows that binding sites for whole nucleotides can be effectively reconstructed by assembling modules, which are themselves predicted from non-homologous proteins. Figure 4 reports a test case involving a FAD binding site on subunit F of the Alkyl hydroperoxide reductase from *S. typhymurium* (PDB code 1HYU).

### Comparison with an Existing Method

We compared our approach with a method developed by Saito et al. [17] aimed at predicting nucleotide-binding sites by identifying structural motifs for the nucleotide bases. This method uses empirical scores derived from a set of non-redundant nucleotide-protein complexes, divided into four sets: adenine-, guanine-, nicotinamide- and flavin-containing nucleotides. The authors derived an empirical scoring system from a learning dataset of proteins. This score is associated to the spatial distribution of protein atoms around the nucleobase moiety in a 12 Å radius, so that the frequencies of 13 types of protein atoms are calculated. When analyzing a protein structure this system assigns a score to each point of the grid in which the protein is plunged, and then it clusters overlapping predicted nucleobases. Finally the method ranks the predictions and retains the best 100 considering them as correct if they have a R.M.S.D. with the known binding site equal or lower than 3 Å. We evaluated predicted nucleobases in the same way, selecting from the cluster of our prediction a nucleobase identical to the one bound by the protein.

In order to compare the two methods we took the dataset used to test the method developed by Saito, composed of 380 protein structures divided into four sets: structures binding adenine-, guanine-, nicotinamide- and flavin- containing molecules. We excluded from the analysis 40 proteins that do not bind any nucleotide. Moreover 36 proteins do not have sufficient data for the calculation of the conservation of their residues (see the “Scoring of the predicted nucleotide-binding sites” paragraph in the “Methods“ section) or the software for the calculation of the protein solvent excluded surface produced an error (see the “Filtering and clustering of the predicted nucleotide modules” in the “Methods” section). The final dataset is composed of 306 protein structures. Our method performs better both on average on all nucleotides and on specific nucleotide sets: considering the first-ranked prediction their method has a performance of 31%, 29%, 32% and 40% while our method reaches a performance of 32%, 46%, 55% and 47% on adenine, guanine, nicotinamide and flavin respectively. Saito et al.’s method is focused on the nucleobase moiety, while we demonstrated that with some nucleotides, such as the ones containing nicotinamide, the carbohydrate module is more effective in predicting the nucleotide-binding site. Therefore we also used the carbohydrate and the phosphate modules to predict nucleotide-binding sites on the same set of proteins. Our method still predicts nucleotide-binding sites better then Saito’s method and remarkably shows that the carbohydrate reaches a performance of 72% and 63% respectively in the nicotinamide and flavin set of proteins. Complete results are in Figure 5.

### Discrimination between Protein Structures Binding/not-binding Nucleotides

We tested our method for its ability to discriminate nucleotide-binding proteins from proteins that do not bind nucleotides. In order to properly test the method we built a “negative” dataset of protein structures that do not bind nucleotides containing the same number of structures of the sc-PDB dataset. The structures were obtained from BLASTClust homology-based groups of protein chains (non-redundant at 30% of sequence identity), by randomly choosing 924 groups that do not include any structures binding a nucleotide. For each group we selected a random protein chain. When predicting nucleotide-binding sites the minimum allowed sub-architecture is composed of two consecutive nucleotide modules (nucleobase-carbohydrate or carbohydrate-phosphate). We used the same criterion for this test. For each analyzed protein we selected only the top-scoring prediction and assigned its score to the protein.

We did a tenfold cross-validation test using a 9∶1 ratio for the training and test sets. In the training phase we identified the optimal scoring threshold as 166.33. This threshold was found to better discriminate the two classes of structures (average Matthews Correlation Coefficient of 0.6). The chosen threshold was then applied to the corresponding test sets. The average MCC achieved by our method during the cross-validation was 0.6, with an average sensitivity of 0.64 and specificity of 0.93.

### Application of the Method on Homology Models

One of the main problems of protein structural analysis is the limited amount of available structures, even if their number is increasing at a fast pace. In order to increase the application range of our method we evaluated its performance on homology models. We culled a set of 110 protein structures from the sc-PDB dataset by selecting 10 random structures for each of the eleven nucleotide types considered in this work.

We searched template structures for each of the proteins in this dataset, using BLAST with the following thresholds: sequence identity between 30% and 99% and maximum e-value of 10e−6. We used Modeller (v.9.9) [33] to build 942 homology models with all the template structures identified, and calculated the Global Distance Test (GDT) [34] score for each model. We used the method described in this work to predict nucleotide binding sites and evaluated its performance as a function of the GDT score of the model. To evaluate the correctness of the prediction each model was superimposed on the query protein. The performance (percentage of proteins with top-ranked correct predictions) drops from 63% to 42% when going from the PDB structure to the models with the highest GDT (see Figure 6). Therefore, even when using the best homology models, there are structural variations that impair the correct prediction of nucleotide-binding sites. However the performance of the method remains higher than 35% for GDT values over 50, thus demonstrating that these models can be effectively used for prediction.

## Conclusions

In this work we developed a new method to predict nucleotide-binding sites in protein structures. In particular we investigated whether the concept of modularity [16] can be used to reconstruct nucleotide-binding sites starting from the predicted locations of their constituent modules. The method compares a query protein structure with a dataset of template binding sites for nucleotide modules, namely the nucleobase, the carbohydrate and the phosphate. The predicted binding sites are evaluated by considering their distribution on the protein surface and the conservation of the residues that identify their binding site. The method has been tested on a set of 924 non-redundant protein structures binding eleven types of nucleotides. We performed a cross-validation in order to find the combination of parameters that provides the optimal scoring threshold; the method reaches F-scores of 0.48, 0.47 and 0.64 when predicting nucleobases, carbohydrates and phosphates respectively.

We also considered the performance of the method in ranking the predictions irrespective of any scoring threshold. The method ranks as first a prediction that identifies the nucleotide-binding site in 48%, 48% and 68% of protein structures for the nucleobase, carbohydrate and phosphate respectively. When considering the first five ranks the method reaches a performance of 71%, 65% and 86%. Our results show that the phosphate module is the one that, on average, better identifies nucleotide-binding sites, while the two other modules perform better on specific nucleotides, like FAD NAD and NAP. Nearly half of the correct first ranked predictions for nucleobases and carbohydrates and the majority of them for the phosphates involve structural similarity between different folds, confirming the outcome of previous studies and the promiscuity of phosphate binding sites.

Moreover the method has been tested on a set of 64 non-redundant proteins whose structure has been solved both with and without the nucleotide. This was done to test the ability of the method in a real world case, where the structure has been solved in the unbound state. The performance decreases by 7% when considering only the first ranked predictions, showing that the method is reliable in the annotation of new structures solved in the unbound state.

We also investigated the possibility of combining predicted binding sites for different nucleotide modules with the aim to reconstruct the whole nucleotide-binding site, or part of it, from the predictions. After scoring and ranking the predictions the method ranks as first a correct prediction in 59% of the proteins tested. This demonstrates that the predicted module positions can be effectively extended to reconstruct the structure of the nucleotide-binding site. Moreover we saw that in 98% of these successful cases each module was bound by a non-homologous protein chain, thus demonstrating that binding sites can be predicted on proteins with novel folds. Our approach has a superior performance when compared with an existing method that predicts nucleotide-binding sites using the position of the nucleobase moiety. Moreover we show that in some cases other nucleotide modules are better than the nucleobase in identifying the binding site. For instance the carbohydrate is better than the nucleobase in identifying binding sites for nicotinamide-containing nucleotides. Our results show that the present method can be a useful resource in functional annotation of nucleotide-binding proteins when homology-based approaches fail. Our method does not try to build the conformation of the nucleotide molecule. Rather, the aim is to pinpoint the location of the binding site, thus providing a useful starting point for other methodologies such as protein-ligand docking. The method is available and downloadable at http://pdbfun.uniroma2.it/nucleos.

## Supporting Information

Table S1 **List of the 924 protein structures included in the sc-PDB dataset.** The table reports the PDB code, the chain of the protein structure analyzed by the method, the nucleotide bound by the protein, the protein name and name of the organism. Proteins are grouped by the type of nucleotide bound.
(DOCX)

Table S2 **List of the 64 protein structures included in the LigASite dataset.** The table reports the PDB code of the apo and holo structures (grouped by the type of nucleotide bound) of the same protein, the chain of the protein structures analyzed by the method, the nucleotide bound by the protein, the protein name and name of the organism.
(DOCX)
