# Supplementary material for: Identification of Nucleotide-Binding Sites in Protein Structures: A Novel Approach Based on Nucleotide Modularity
Source: PLoS One. 2012 Nov 27;7(11):e50240. doi: 10.1371/journal.pone.0050240 (PMC3507729; doi:10.1371/journal.pone.0050240)
Supplement: Table S1 — List of the 924 protein structures included in the sc-PDB dataset. The table reports the PDB code, the chain of the protein structure analyzed by the method, the nucleotide bound by the protein, the protein name and name of the organism. Proteins are grouped by the type of nucleotide bound. (DOCX) [file pone.0050240.s001.docx]

| **PDB** | **Chain** | **Ligand** | **Protein name** | **Species** |
| --- | --- | --- | --- | --- |
| 1hsk | A | FAD | UDP-N-ACETYLENOLPYRUVOYLGLUCOSAMINE REDUCTASE | Staphylococcus aureus |
| 3crz | A | FAD | Ferredoxin--NADP+ reductase | Pseudomonas aeruginosa |
| 2hti | A | FAD | BH0577 protein | Bacillus halodurans |
| 1hyu | A | FAD | ALKYL HYDROPEROXIDE REDUCTASE SUBUNIT F | Salmonella enterica subsp. enterica serovar Typhimurium |
| 3lov | A | FAD | Protoporphyrinogen oxidase | Exiguobacterium sibiricum 255-15 |
| 1rm6 | B | FAD | 4-hydroxybenzoyl-CoA reductase alpha subunit | Thauera aromatica |
| 3g5q | A | FAD | Methylenetetrahydrofolate--tRNA-(uracil-5-)-methyltransferase trmFO | Thermus thermophilus HB8 |
| 1ybh | A | FAD | Acetolactate synthase, chloroplast | Arabidopsis thaliana |
| 3f8d | C | FAD | Thioredoxin reductase (TrxB-3) | Sulfolobus solfataricus |
| 1ddg | B | FAD | SULFITE REDUCTASE (NADPH) FLAVOPROTEIN ALPHA-COMPONENT | Escherichia coli |
| 3fst | A | FAD | 5,10-methylenetetrahydrofolate reductase | Escherichia coli K-12 |
| 2cul | A | FAD | Glucose-inhibited division protein A-related protein, probable oxidoreductase | Thermus thermophilus HB8 |
| 3grs | A | FAD | GLUTATHIONE REDUCTASE | Homo sapiens |
| 2vou | A | FAD | 2,6-DIHYDROXYPYRIDINE HYDROXYLASE | Arthrobacter nicotinovorans |
| 3lcm | B | FAD | Putative oxidoreductase | Streptococcus mutans UA159 |
| 2e3t | A | FAD | Xanthine dehydrogenase/oxidase | Rattus norvegicus |
| 2aqj | A | FAD | tryptophan halogenase, PrnA | Pseudomonas fluorescens |
| 3kpf | A | FAD | Polyamine oxidase | Zea mays |
| 3pvc | A | FAD | tRNA 5-methylaminomethyl-2-thiouridine biosynthesis bifunctional protein mnmC | Yersinia pestis |
| 3ewk | A | FAD | Sensor protein | Methylococcus capsulatus |
| 3fbs | B | FAD | Oxidoreductase | Agrobacterium tumefaciens str. C58 |
| 3gmb | A | FAD | 2-methyl-3-hydroxypyridine-5-carboxylic acid oxygenase | Mesorhizobium loti |
| 2r0c | A | FAD | RebC | Lechevalieria aerocolonigenes |
| 3dme | A | FAD | conserved exported protein | Bordetella pertussis |
| 1m6i | A | FAD | Programmed cell death protein 8 | Homo sapiens |
| 2qwx | B | FAD | Ribosyldihydronicotinamide dehydrogenase [quinone] | Homo sapiens |
| 3e2q | A | FAD | Proline dehydrogenase | Escherichia coli K-12 |
| 3pm9 | D | FAD | Putative oxidoreductase | Rhodopseudomonas palustris |
| 1jnr | A | FAD | adenylylsulfate reductase | Archaeoglobus fulgidus DSM 4304 |
| 3m0o | A | FAD | Monomeric sarcosine oxidase | Bacillus sp. B-0618 |
| 2wq7 | A | FAD | RE11660P | Drosophila melanogaster |
| 2ylz | A | FAD | PHENYLACETONE MONOOXYGENASE | Thermobifida fusca |
| 1n4w | A | FAD | Cholesterol oxidase | Streptomyces sp. SA-COO |
| 2r6h | C | FAD | NADH:ubiquinone oxidoreductase, Na translocating, F subunit | Porphyromonas gingivalis W83 |
| 3rpe | A | FAD | Modulator of drug activity B | Yersinia pestis |
| 2d29 | A | FAD | acyl-CoA dehydrogenase | Thermus thermophilus HB8 |
| 3ahq | A | FAD | ERO1-like protein alpha | Homo sapiens |
| 3hzg | D | FAD | Thymidylate synthase thyX | Mycobacterium tuberculosis |
| 2vfs | A | FAD | XYLITOL OXIDASE | Streptomyces coelicolor A3(2) |
| 2xve | C | FAD | FLAVIN-CONTAINING MONOOXYGENASE | Methylophaga aminisulfidivorans |
| 2cnd | A | FAD | NADH-DEPENDENT NITRATE REDUCTASE | Zea mays |
| 3mbg | A | FAD | FAD-linked sulfhydryl oxidase ALR | Homo sapiens |
| 2gmj | B | FAD | Electron transfer flavoprotein-ubiquinone oxidoreductase | Sus scrofa |
| 1owl | A | FAD | Deoxyribodipyrimidine photolyase | Synechococcus elongatus PCC 6301 |
| 2dki | A | FAD | 3-HYDROXYBENZOATE HYDROXYLASE | Comamonas testosteroni |
| 2i0k | A | FAD | Oxidoreductase | Brevibacterium sterolicum |
| 3mkh | C | FAD | NITROALKANE OXIDASE | Podospora anserina |
| 3k87 | B | FAD | Chlorophenol-4-monooxygenase component 1 | Burkholderia cepacia |
| 3m31 | A | FAD | Endoplasmic oxidoreductin-1 | Saccharomyces cerevisiae |
| 1krh | A | FAD | Benzoate 1,2-Dioxygenase Reductase | Acinetobacter sp. |
| 2gqf | A | FAD | Hypothetical protein HI0933 | Haemophilus influenzae |
| 2x3n | A | FAD | PROBABLE FAD-DEPENDENT MONOOXYGENASE | Pseudomonas aeruginosa |
| 3kkj | A | FAD | Amine oxidase, flavin-containing | Pseudomonas syringae pv. tomato |
| 1f0x | A | FAD | D-LACTATE DEHYDROGENASE | Escherichia coli |
| 2rgh | A | FAD | Alpha-Glycerophosphate Oxidase | Streptococcus sp. |
| 3ic9 | B | FAD | dihydrolipoamide dehydrogenase | Colwellia psychrerythraea 34H |
| 1u8v | A | FAD | Gamma-aminobutyrate metabolism dehydratase/isomerase | Clostridium aminobutyricum |
| 3llk | C | FAD | Sulfhydryl oxidase 1 | Homo sapiens |
| 2bi7 | A | FAD | UDP-GALACTOPYRANOSE MUTASE | Klebsiella pneumoniae |
| 3gwn | B | FAD | Probable FAD-linked sulfhydryl oxidase R596 | Acanthamoeba polyphaga mimivirus |
| 3pl8 | A | FAD | Pyranose 2-oxidase | Trametes ochracea |
| 2qtz | A | FAD | Methionine synthase reductase | Homo sapiens |
| 1qlt | A | FAD | VANILLYL-ALCOHOL OXIDASE | Penicillium simplicissimum |
| 2yr5 | A | FAD | Pro-enzyme of L-phenylalanine oxidase | Pseudomonas sp. P-501 |
| 2bk3 | A | FAD | AMINE OXIDASE [FLAVIN-CONTAINING] B | Homo sapiens |
| 1fdr | A | FAD | FLAVODOXIN REDUCTASE | Escherichia coli |
| 2e5v | A | FAD | L-aspartate oxidase | Sulfolobus tokodaii |
| 2qcu | A | FAD | Aerobic glycerol-3-phosphate dehydrogenase | Escherichia coli |
| 2vvl | B | FAD | MONOAMINE OXIDASE N | Aspergillus niger |
| 2e1m | A | FAD | L-glutamate oxidase | Streptomyces sp. X-119-6 |
| 3owa | B | FAD | Acyl-CoA dehydrogenase | Bacillus anthracis str. 'Ames Ancestor' |
| 3qvp | A | FAD | Glucose oxidase | Aspergillus niger |
| 3n0b | C | FAD | Thymidylate synthase thyX | Thermotoga maritima |
| 2dji | A | FAD | Pyruvate oxidase | Aerococcus viridans |
| 3d1c | A | FAD | Flavin-containing Putative Monooxygenase | Staphylococcus aureus subsp. aureus Mu50 |
| 1k0l | A | FAD | P-HYDROXYBENZOATE HYDROXYLASE | Pseudomonas aeruginosa |
| 1ju2 | A | FAD | hydroxynitrile lyase | Prunus dulcis |
| 2bry | B | FAD | NEDD9 INTERACTING PROTEIN WITH CALPONIN HOMOLOGY AND LIM DOMAINS | Mus musculus |
| 2qa1 | A | FAD | Polyketide oxygenase PgaE | Streptomyces sp. PGA64 |
| 3kpk | A | FAD | Sulfide-quinone reductase, putative | Acidithiobacillus ferrooxidans ATCC 23270 |
| 2yg5 | A | FAD | PUTRESCINE OXIDASE | Rhodococcus erythropolis |
| 1w07 | A | FAD | ACYL-COA OXIDASE | Arabidopsis thaliana |
| 3ics | A | FAD | Coenzyme A-Disulfide Reductase | Bacillus anthracis str. Ames |
| 3djl | A | FAD | Protein aidB | Escherichia coli |
| 2q4w | A | FAD | Cytokinin dehydrogenase 7 | Arabidopsis thaliana |
| 1mo9 | A | FAD | orf3 | Xanthobacter autotrophicus Py2 |
| 3gwl | B | FAD | FAD-linked sulfhydryl oxidase | African swine fever virus BA71V |
| 3oc4 | B | FAD | Oxidoreductase, pyridine nucleotide-disulfide family | Enterococcus faecalis |
| 1cjc | A | FAD | PROTEIN (ADRENODOXIN REDUCTASE) | Bos taurus |
| 3nye | A | FAD | D-Arginine Dehydrogenase | Pseudomonas aeruginosa |
| 1ng4 | A | FAD | Glycine oxidase | Bacillus subtilis |
| 1nhp | A | FAD | NADH PEROXIDASE | Enterococcus faecalis |
| 3nix | H | FAD | Flavoprotein/dehydrogenase | Cytophaga hutchinsonii ATCC 33406 |
| 2x8g | A | FAD | THIOREDOXIN GLUTATHIONE REDUCTASE | Schistosoma mansoni |
| 3lo8 | A | FAD | Ferredoxin--NADP reductase | Zea mays |
| 3ad7 | B | FAD | Subunit alpha of sarcosine oxidase | Corynebacterium sp. U-96 |
| 2wbi | A | FAD | ACYL-COA DEHYDROGENASE FAMILY MEMBER 11 | Homo sapiens |
| 3n3y | D | FAD | Thymidylate synthase thyX | Helicobacter pylori SS1 |
| 1jr8 | A | FAD | Erv2 PROTEIN, mitochondrial | Saccharomyces cerevisiae |
| 3ka7 | A | FAD | Oxidoreductase | Methanosarcina mazei |
| 2zxi | D | FAD | tRNA uridine 5-carboxymethylaminomethyl modification enzyme mnmG | Aquifex aeolicus |
| 3fmw | A | FAD | Oxygenase | Streptomyces argillaceus |
| 3i99 | A | FAD | UDP-N-acetylenolpyruvoylglucosamine reductase | Vibrio cholerae O1 biovar El Tor str. N16961 |
| 1gte | A | FAD | DIHYDROPYRIMIDINE DEHYDROGENASE | Sus scrofa |
| 3pnd | D | FAD | Thiamine biosynthesis lipoprotein ApbE | Salmonella enterica subsp. enterica serovar Typhimurium |
| 1oqc | D | FAD | augmenter of liver regeneration | Rattus norvegicus |
| 1rsg | A | FAD | FMS1 protein | Saccharomyces cerevisiae |
| 2rgj | A | FAD | Flavin-containing monooxygenase | Pseudomonas aeruginosa |
| 2gqw | A | FAD | ferredoxin reductase | Pseudomonas sp. KKS102 |
| 1ve9 | B | FAD | D-amino acid oxidase | Sus scrofa |
| 2xdo | D | FAD | TETX2 PROTEIN | Bacteroides thetaiotaomicron |
| 3nlc | A | FAD | Uncharacterized protein VP0956 | Vibrio parahaemolyticus |
| 2ywl | B | FAD | Thioredoxin reductase related protein | Thermus thermophilus HB8 |
| 3g6k | C | FAD | FMN adenylyltransferase | Candida glabrata |
| 1n2s | A | NAD | dTDP-glucose oxidoreductase | Salmonella enterica subsp. enterica serovar Typhimurium |
| 1gzf | A | NAD | MONO-ADP-RIBOSYLTRANSFERASE C3 | Clostridium botulinum |
| 2ekp | A | NAD | 2-deoxy-D-gluconate 3-dehydrogenase | Thermus thermophilus HB8 |
| 1lj8 | A | NAD | mannitol dehydrogenase | Pseudomonas fluorescens |
| 3p2o | B | NAD | Bifunctional protein folD | Campylobacter jejuni subsp. jejuni NCTC 11168 = ATCC 700819 |
| 1lc3 | A | NAD | Biliverdin Reductase A | Rattus norvegicus |
| 1o04 | A | NAD | Aldehyde dehydrogenase, mitochondrial precursor | Homo sapiens |
| 3lvf | Q | NAD | Glyceraldehyde-3-phosphate dehydrogenase 1 | Staphylococcus aureus subsp. aureus MRSA252 |
| 2xxj | B | NAD | L-LACTATE DEHYDROGENASE | Thermus thermophilus HB8 |
| 3f3s | A | NAD | Lambda-crystallin homolog | Homo sapiens |
| 1mi3 | B | NAD | xylose reductase | Candida tenuis |
| 2jhf | B | NAD | ALCOHOL DEHYDROGENASE E CHAIN | Equus caballus |
| 2czc | A | NAD | Glyceraldehyde-3-phosphate dehydrogenase | Pyrococcus horikoshii OT3 |
| 2ph5 | A | NAD | Homospermidine synthase | Legionella pneumophila subsp. pneumophila str. Philadelphia 1 |
| 3pvz | C | NAD | UDP-N-acetylglucosamine 4,6-dehydratase | Vibrio fischeri ES114 |
| 1ebf | A | NAD | HOMOSERINE DEHYDROGENASE | Saccharomyces cerevisiae |
| 1kol | A | NAD | formaldehyde dehydrogenase | Pseudomonas putida |
| 3flk | D | NAD | Tartrate dehydrogenase/decarboxylase | Pseudomonas putida |
| 1wdk | A | NAD | Fatty oxidation complex alpha subunit | Pseudomonas fragi |
| 3m6i | A | NAD | L-arabinitol 4-dehydrogenase | Neurospora crassa |
| 3e18 | A | NAD | oxidoreductase | Listeria innocua |
| 3l98 | A | NAD | Citrate synthase | Escherichia coli |
| 2gsd | A | NAD | NAD-dependent formate dehydrogenase | Moraxella sp. |
| 1dru | A | NAD | DIHYDRODIPICOLINATE REDUCTASE | Escherichia coli |
| 2g5c | A | NAD | prephenate dehydrogenase | Aquifex aeolicus VF5 |
| 1ej2 | A | NAD | NICOTINAMIDE MONONUCLEOTIDE ADENYLYLTRANSFERASE | Methanothermobacter thermautotrophicus |
| 1wxh | A | NAD | NH(3)-dependent NAD(+) synthetase | Escherichia coli |
| 3rfx | C | NAD | Uronate dehydrogenase | Agrobacterium tumefaciens str. C58 |
| 2pzj | A | NAD | Putative nucleotide sugar epimerase/ dehydratase | Bordetella bronchiseptica |
| 1bmd | A | NAD | MALATE DEHYDROGENASE | Thermus thermophilus |
| 1ek5 | A | NAD | UDP-GALACTOSE 4-EPIMERASE | Homo sapiens |
| 1f0y | A | NAD | L-3-HYDROXYACYL-COA DEHYDROGENASE | Homo sapiens |
| 1u1i | A | NAD | myo-inositol-1-phosphate synthase | Archaeoglobus fulgidus DSM 4304 |
| 3b82 | F | NAD | Elongation factor 2 | Saccharomyces cerevisiae |
| 3cin | A | NAD | Myo-inositol-1-phosphate synthase-related protein | Thermotoga maritima MSB8 |
| 2i65 | A | NAD | ADP-ribosyl cyclase 1 | Homo sapiens |
| 1j5p | A | NAD | ASPARTATE DEHYDROGENASE | Thermotoga maritima |
| 1mg5 | A | NAD | alcohol dehydrogenase | Drosophila melanogaster |
| 3keo | A | NAD | Redox-sensing transcriptional repressor rex | Streptococcus agalactiae serogroup III |
| 1x14 | B | NAD | NAD(P) transhydrogenase subunit alpha | Escherichia coli |
| 1ee9 | A | NAD | 5,10-METHYLENETETRAHYDROFOLATE DEHYDROGENASE | Saccharomyces cerevisiae |
| 1p1h | D | NAD | Inositol-3-phosphate synthase | Saccharomyces cerevisiae |
| 1sg6 | A | NAD | Pentafunctional AROM polypeptide | Emericella nidulans |
| 1gr0 | A | NAD | INOSITOL-3-PHOSPHATE SYNTHASE | Mycobacterium tuberculosis |
| 1lss | C | NAD | Trk system potassium uptake protein trkA homolog | Methanocaldococcus jannaschii |
| 3ojl | B | NAD | Cap5O | Staphylococcus aureus |
| 1i2b | A | NAD | SULFOLIPID BIOSYNTHESIS PROTEIN SQD1 | Arabidopsis thaliana |
| 2o4c | A | NAD | Erythronate-4-phosphate dehydrogenase | Pseudomonas aeruginosa |
| 2pla | B | NAD | Glycerol-3-phosphate dehydrogenase 1-like protein | Homo sapiens |
| 1c1d | A | NAD | L-PHENYLALANINE DEHYDROGENASE | Rhodococcus sp. |
| 1evj | A | NAD | GLUCOSE-FRUCTOSE OXIDOREDUCTASE | Zymomonas mobilis |
| 3c7a | A | NAD | Octopine dehydrogenase | Pecten maximus |
| 1s20 | A | NAD | Hypothetical oxidoreductase yiaK | Escherichia coli |
| 1zbq | B | NAD | 17-beta-hydroxysteroid dehydrogenase 4 | Homo sapiens |
| 2izz | A | NAD | PYRROLINE-5-CARBOXYLATE REDUCTASE 1 | Homo sapiens |
| 1kqn | B | NAD | NICOTINAMIDE MONONUCLEOTIDE ADENYLYL TRANSFERASE | Homo sapiens |
| 3ajr | A | NAD | NDP-sugar epimerase | Thermoplasma volcanium GSS1 |
| 2dvm | D | NAD | 439aa long hypothetical malate oxidoreductase | Pyrococcus horikoshii |
| 1p9l | A | NAD | dihydrodipicolinate reductase | Mycobacterium tuberculosis |
| 2ekl | A | NAD | D-3-phosphoglycerate dehydrogenase | Sulfolobus tokodaii |
| 3q3c | A | NAD | Probable 3-hydroxyisobutyrate dehydrogenase | Pseudomonas aeruginosa |
| 1jq5 | A | NAD | Glycerol dehydrogenase | Geobacillus stearothermophilus |
| 1h94 | A | NAD | GLUCOSE 6-PHOSPHATE 1-DEHYDROGENASE | Leuconostoc mesenteroides |
| 1uwl | A | NAD | UROCANATE HYDRATASE | Pseudomonas putida |
| 1qr6 | A | NAD | MALIC ENZYME 2 | Homo sapiens |
| 1ici | A | NAD | TRANSCRIPTIONAL REGULATORY PROTEIN, SIR2 FAMILY | Archaeoglobus fulgidus |
| 3jyo | A | NAD | Quinate/shikimate dehydrogenase | Corynebacterium glutamicum ATCC 13032 |
| 1vbi | A | NAD | Type 2 malate/lactate dehydrogenase | Thermus thermophilus |
| 3oa2 | B | NAD | WbpB | Pseudomonas aeruginosa |
| 1rlz | A | NAD | Deoxyhypusine synthase | Homo sapiens |
| 1u7h | B | NAD | ornithine cyclodeaminase | Pseudomonas putida |
| 3hl0 | A | NAD | Maleylacetate reductase | Agrobacterium tumefaciens str. C58 |
| 1rkx | B | NAD | CDP-glucose-4,6-dehydratase | Yersinia pseudotuberculosis |
| 2i2f | A | NAD | Probable inorganic polyphosphate/ATP-NAD kinase 1 | Listeria monocytogenes |
| 2w2l | A | NAD | D-MANDELATE DEHYDROGENASE | Rhodotorula graminis |
| 1nuu | B | NAD | FKSG76 | Homo sapiens |
| 1dhr | A | NAD | DIHYDROPTERIDINE REDUCTASE | Rattus norvegicus |
| 1xah | B | NAD | 3-dehydroquinate synthase | Staphylococcus aureus |
| 3bts | B | NAD | Galactose/lactose metabolism regulatory protein GAL80 | Saccharomyces cerevisiae |
| 1sc6 | D | NAD | D-3-phosphoglycerate dehydrogenase | Escherichia coli |
| 1omo | A | NAD | alanine dehydrogenase | Archaeoglobus fulgidus |
| 3ec7 | B | NAD | Putative Dehydrogenase | Salmonella enterica subsp. enterica serovar Typhimurium str. LT2 |
| 3sc6 | A | NAP | dTDP-4-dehydrorhamnose reductase | Bacillus anthracis str. Ames |
| 1h5q | B | NAP | NADP-DEPENDENT MANNITOL DEHYDROGENASE | Agaricus bisporus |
| 3dtt | A | NAP | NADP oxidoreductase | Arthrobacter sp. FB24 |
| 2v6g | A | NAP | PROGESTERONE 5-BETA-REDUCTASE | Digitalis lanata |
| 1ez0 | C | NAP | ALDEHYDE DEHYDROGENASE | Vibrio harveyi |
| 3lns | A | NAP | Benzaldehyde dehydrogenase | Pseudomonas putida |
| 3ngl | C | NAP | Bifunctional protein folD | Thermoplasma acidophilum |
| 2h63 | B | NAP | Biliverdin reductase A | Homo sapiens |
| 3baz | A | NAP | Hydroxyphenylpyruvate reductase | Solenostemon scutellarioides |
| 2ehq | A | NAP | 1-pyrroline-5-carboxylate dehydrogenase | Thermus thermophilus HB8 |
| 3b70 | A | NAP | Enoyl reductase | Aspergillus terreus |
| 3bhi | A | NAP | Carbonyl reductase [NADPH] 1 | Homo sapiens |
| 2jl1 | A | NAP | TRIPHENYLMETHANE REDUCTASE | Citrobacter sp. MY-5 |
| 1pno | A | NAP | NAD(P) transhydrogenase subunit beta | Rhodospirillum rubrum |
| 2xnj | A | NAP | FERREDOXIN NADP-H REDUCTASE | Escherichia coli |
| 1qyv | A | NAP | Estradiol 17 beta-dehydrogenase 1 | Homo sapiens |
| 2vna | A | NAP | PROSTAGLANDIN REDUCTASE 2 | Homo sapiens |
| 2yut | A | NAP | Putative short-chain oxidoreductase | Thermus thermophilus HB8 |
| 1z0u | B | NAP | Probable inorganic polyphosphate/ATP-NAD kinase | Archaeoglobus fulgidus |
| 2y53 | A | NAP | ALDEHYDE DEHYDROGENASE (BOX PATHWAY) | Burkholderia xenovorans LB400 |
| 1djl | B | NAP | TRANSHYDROGENASE DIII | Homo sapiens |
| 2ahr | D | NAP | putative pyrroline carboxylate reductase | Streptococcus pyogenes M1 GAS |
| 1h9a | A | NAP | GLUCOSE 6-PHOSPHATE 1-DEHYDROGENASE | Leuconostoc mesenteroides |
| 1hye | A | NAP | L-LACTATE/MALATE DEHYDROGENASE | Methanocaldococcus jannaschii |
| 1og6 | A | NAP | HYPOTHETICAL OXIDOREDUCTASE YDHF | Escherichia coli K-12 |
| 3d84 | X | NAP | Dihydrofolate reductase | Mus musculus |
| 2vq3 | B | NAP | METALLOREDUCTASE STEAP3 | Homo sapiens |
| 2gz1 | B | NAP | Aspartate beta-semialdehyde dehydrogenase | Streptococcus pneumoniae |
| 2yyy | A | NAP | Glyceraldehyde-3-phosphate dehydrogenase | Methanocaldococcus jannaschii DSM 2661 |
| 1nyt | A | NAP | Shikimate 5-dehydrogenase | Escherichia coli |
| 2raf | A | NAP | Putative Dinucleotide-Binding Oxidoreductase | Lactobacillus plantarum WCFS1 |
| 1z8a | A | NAP | aldose reductase | Homo sapiens |
| 2c0c | A | NAP | ZINC BINDING ALCOHOL DEHYDROGENASE, DOMAIN CONTAINING 2 | Homo sapiens |
| 2cmj | A | NAP | ISOCITRATE DEHYDROGENASE [NADP] CYTOPLASMIC | Mus musculus |
| 1lua | A | NAP | Methylene Tetrahydromethanopterin Dehydrogenase | Methylobacterium extorquens AM1 |
| 1xq6 | A | NAP | unknown protein | Arabidopsis thaliana |
| 2o7p | A | NAP | Riboflavin biosynthesis protein ribD | Escherichia coli K-12 |
| 2f1k | C | NAP | prephenate dehydrogenase | Synechocystis sp. PCC 6803 |
| 1w6u | C | NAP | 2,4-DIENOYL-COA REDUCTASE, MITOCHONDRIAL PRECURSOR | Homo sapiens |
| 3e8x | A | NAP | Putative NAD-dependent epimerase/dehydratase | Bacillus halodurans |
| 1zsx | A | NAP | Voltage-gated potassium channel beta-2 subunit | Homo sapiens |
| 3nx4 | A | NAP | Putative oxidoreductase | Salmonella enterica subsp. enterica serovar Typhimurium str. LT2 |
| 1sny | A | NAP | sniffer CG10964-PA | Drosophila melanogaster |
| 2rir | B | NAP | Dipicolinate synthase, A chain | Bacillus subtilis |
| 1pqu | D | NAP | Aspartate-semialdehyde dehydrogenase | Haemophilus influenzae Rd KW20 |
| 1zh8 | B | NAP | oxidoreductase | Thermotoga maritima MSB8 |
| 2i2a | A | NAP | Probable inorganic polyphosphate/ATP-NAD kinase 1 | Listeria monocytogenes |
| 1sep | A | NAP | SEPIAPTERIN REDUCTASE | Mus musculus |
| 1vhd | A | NAP | alcohol dehydrogenase, iron-containing | Thermotoga maritima |
| 3rbv | A | NAP | Sugar 3-ketoreductase | Actinomadura kijaniata |
| 2ag8 | A | NAP | pyrroline-5-carboxylate reductase | Neisseria meningitidis MC58 |
| 1hdo | A | NAP | BILIVERDIN IX BETA REDUCTASE | Homo sapiens |
| 2i3g | A | NAP | N-acetyl-gamma-glutamyl-phosphate reductase | Mycobacterium tuberculosis H37Rv |
| 1yjq | A | NAP | 2-dehydropantoate 2-reductase | Escherichia coli |
| 2qw8 | B | NAP | Eugenol synthase 1 | Ocimum basilicum |
| 1ykf | A | NAP | NADP-DEPENDENT ALCOHOL DEHYDROGENASE | Thermoanaerobacter brockii |
| 1fxs | A | NAP | PROTEIN (GDP-FUCOSE SYNTHETASE) | Escherichia coli K-12 |
| 1ai2 | A | NAP | ISOCITRATE DEHYDROGENASE | Escherichia coli |
| 3mbc | A | NAP | Isocitrate dehydrogenase [NADP] | Corynebacterium glutamicum ATCC 13032 |
| 2r01 | A | FMN | Nitroreductase family protein | Chlorobium tepidum TLS |
| 3b6i | A | FMN | Flavoprotein wrbA | Escherichia coli K-12 |
| 2wqf | A | FMN | COPPER INDUCED NITROREDUCTASE D | Lactococcus lactis |
| 1nrg | A | FMN | pyridoxine 5'-phosphate oxidase | Homo sapiens |
| 1t0i | A | FMN | YLR011wp | Saccharomyces cerevisiae |
| 2pr5 | A | FMN | Blue-light photoreceptor | Bacillus subtilis |
| 1ty9 | A | FMN | Phenazine biosynthesis protein phzG | Pseudomonas fluorescens |
| 3e10 | A | FMN | Putative NADH Oxidase | Clostridium acetobutylicum |
| 1i0r | A | FMN | CONSERVED HYPOTHETICAL PROTEIN | Archaeoglobus fulgidus |
| 3gfa | A | FMN | Putative nitroreductase | Clostridium difficile 630 |
| 1t5b | A | FMN | Acyl carrier protein phosphodiesterase | Salmonella enterica subsp. enterica serovar Typhimurium |
| 3k30 | A | FMN | Histamine dehydrogenase | Pimelobacter simplex |
| 3b9o | B | FMN | Alkane monooxygenase | Geobacillus thermodenitrificans |
| 1obo | A | FMN | FLAVODOXIN | Nostoc sp. PCC 7119 |
| 2pia | A | FMN | PHTHALATE DIOXYGENASE REDUCTASE | Burkholderia cepacia |
| 2fre | A | FMN | NAD(P)H-flavin oxidoreductase | Agrobacterium tumefaciens str. C58 |
| 3l9x | A | FMN | Glutathione-regulated potassium-efflux system protein kefC, linker, ancillary protein kefF | Escherichia coli K-12 |
| 1f4p | A | FMN | FLAVODOXIN | Desulfovibrio vulgaris |
| 2zru | A | FMN | Isopentenyl-diphosphate delta-isomerase | Sulfolobus shibatae |
| 3eo7 | A | FMN | Putative Nitroreductase | Anabaena variabilis ATCC 29413 |
| 2v0u | A | FMN | NPH1-1 | Avena sativa |
| 1vhn | A | FMN | putative flavin oxidoreductase | Thermotoga maritima |
| 2z6d | B | FMN | Phototropin-2 | Arabidopsis thaliana |
| 3pxv | A | FMN | Nitroreductase | Desulfitobacterium hafniense DCB-2 |
| 2prm | A | FMN | Dihydroorotate dehydrogenase, mitochondrial | Homo sapiens |
| 1mvl | A | FMN | PPC decarboxylase AtHAL3a | Arabidopsis thaliana |
| 3of4 | A | FMN | Nitroreductase | Idiomarina loihiensis |
| 2h0u | A | FMN | NADPH-flavin oxidoreductase | Helicobacter pylori |
| 1dnl | A | FMN | PYRIDOXINE 5'-PHOSPHATE OXIDASE | Escherichia coli K-12 |
| 1f5v | A | FMN | OXYGEN-INSENSITIVE NADPH NITROREDUCTASE | Escherichia coli |
| 1nox | A | FMN | NADH OXIDASE | Thermus thermophilus HB8 |
| 2v21 | F | FMN | HYPOTHETICAL PROTEIN TTHA1431 | Thermus thermophilus HB8 |
| 3p7n | B | FMN | Sensor histidine kinase | Erythrobacter litoralis HTCC2594 |
| 3gfs | A | FMN | FMN-dependent NADPH-azoreductase | Bacillus subtilis |
| 3bm1 | A | FMN | Protein ydjA | Escherichia coli K-12 |
| 1eje | A | FMN | FMN-BINDING PROTEIN | Methanothermobacter thermautotrophicus |
| 3gag | C | FMN | Putative NADH dehydrogenase, NADPH nitroreductase | Streptococcus mutans |
| 3qjg | C | FMN | Epidermin biosynthesis protein EpiD | Staphylococcus aureus subsp. aureus COL |
| 3rh7 | F | FMN | Hypothetical oxidoreductase | Sinorhizobium meliloti 1021 |
| 2vzh | A | FMN | NADH-DEPENDENT FMN REDUCTASE | EDTA-degrading bacterium BNC1 |
| 1n9l | A | FMN | putative blue light receptor | Chlamydomonas reinhardtii |
| 2r6v | A | FMN | Uncharacterized protein PH0856 | Pyrococcus horikoshii OT3 |
| 2wzv | A | FMN | NFNB PROTEIN | Mycobacterium smegmatis str. MC2 155 |
| 3e4v | A | FMN | NADH:FMN oxidoreductase like protein | Methylobacillus flagellatus KT |
| 1qzu | C | FMN | hypothetical protein MDS018 | Homo sapiens |
| 1vyr | A | FMN | PENTAERYTHRITOL TETRANITRATE REDUCTASE | Enterobacter cloacae |
| 1vp8 | A | FMN | hypothetical protein AF0103 | Archaeoglobus fulgidus DSM 4304 |
| 3gb5 | A | FMN | Iodotyrosine dehalogenase 1 | Mus musculus |
| 3f2v | A | FMN | General stress protein 14 | Treponema denticola |
| 3koq | A | FMN | NITROREDUCTASE FAMILY PROTEIN | Clostridium difficile 630 |
| 1usc | A | FMN | PUTATIVE STYRENE MONOOXYGENASE SMALL COMPONENT | Thermus thermophilus HB8 |
| 3ek3 | A | FMN | Nitroreductase | Bacteroides fragilis NCTC 9343 |
| 3bw2 | A | FMN | 2-nitropropane dioxygenase | Streptomyces ansochromogenes |
| 3n3a | C | FMN | Ribonucleoside-diphosphate reductase 2 subunit beta | Escherichia coli K-12 |
| 1rlj | A | FMN | NrdI protein | Bacillus subtilis |
| 1flm | A | FMN | PROTEIN (FMN-BINDING PROTEIN) | Desulfovibrio vulgaris str. 'Miyazaki F' |
| 2o12 | A | FMN | Chorismate synthase | Mycobacterium tuberculosis H37Rv |
| 2xod | A | FMN | NRDI PROTEIN | Bacillus anthracis |
| 1ylr | A | FMN | Oxygen-insensitive NAD(P)H nitroreductase | Escherichia coli |
| 3eof | B | FMN | Putative oxidoreductase | Bacteroides fragilis NCTC 9343 |
| 2z6i | A | FMN | Trans-2-enoyl-ACP reductase II | Streptococcus pneumoniae |
| 1sbz | A | FMN | Probable aromatic acid decarboxylase | Escherichia coli O157:H7 |
| 1lm1 | A | FMN | Ferredoxin-dependent glutamate synthase | Synechocystis sp. PCC 6803 |
| 3hr4 | A | FMN | Nitric oxide synthase, inducible | Homo sapiens |
| 2ptf | A | FMN | Uncharacterized protein MTH_863 | Methanothermobacter thermautotrophicus str. Delta H |
| 3bnk | B | FMN | Flavoredoxin | Methanosarcina acetivorans |
| 3gr3 | A | FMN | Nitroreductase | Bartonella henselae str. Houston-1 |
| 3hmz | A | FMN | Flavin reductase domain protein, FMN-binding | Shewanella baltica OS155 |
| 2yab | B | AMP | DEATH-ASSOCIATED PROTEIN KINASE 2 | Mus musculus |
| 3ocv | A | AMP | Lipoprotein E | Haemophilus influenzae |
| 1hdi | A | AMP | PHOSPHOGLYCERATE KINASE | Sus scrofa |
| 3nzt | A | AMP | Glutamate--cysteine ligase | Francisella tularensis subsp. tularensis |
| 3ngt | A | AMP | Nucleoside diphosphate kinase | Leishmania major |
| 1z6s | A | AMP | Ribonuclease pancreatic | Bos taurus |
| 1u9z | A | AMP | Ribose-phosphate pyrophosphokinase | Methanocaldococcus jannaschii |
| 2vii | A | AMP | PSP OPERON TRANSCRIPTIONAL ACTIVATOR | Escherichia coli K-12 |
| 2ze5 | A | AMP | Isopentenyl transferase | Agrobacterium tumefaciens |
| 3omf | A | AMP | Putative histidine triad family protein | Entamoeba histolytica HM-1:IMSS |
| 2yrx | A | AMP | Phosphoribosylglycinamide synthetase | Geobacillus kaustophilus |
| 8gpb | A | AMP | GLYCOGEN PHOSPHORYLASE B | Oryctolagus cuniculus |
| 3ngn | A | AMP | CCR4-NOT transcription complex subunit 6-like | Homo sapiens |
| 2r85 | A | AMP | PurP protein PF1517 | Pyrococcus furiosus |
| 1tbw | B | AMP | Endoplasmin | Canis lupus familiaris |
| 2yvo | A | AMP | MutT/nudix family protein | Thermus thermophilus HB8 |
| 3o0m | A | AMP | HIT family protein | Mycobacterium smegmatis str. MC2 155 |
| 3rk0 | A | AMP | N-type ATP pyrophosphatase superfamily | Pyrococcus furiosus DSM 3638 |
| 3ib8 | A | AMP | Icc protein | Mycobacterium tuberculosis |
| 1son | A | AMP | ADENYLOSUCCINATE SYNTHETASE | Escherichia coli |
| 3lw7 | B | AMP | Adenylate kinase related protein (AdkA-like) | Sulfolobus solfataricus 98/2 |
| 1vd1 | A | AMP | RNase NGR3 | Nicotiana glutinosa |
| 2d1r | A | AMP | Luciferin 4-monooxygenase | Luciola cruciata |
| 2j91 | A | AMP | ADENYLOSUCCINATE LYASE | Homo sapiens |
| 2rh6 | A | AMP | Phosphodiesterase-nucleotide pyrophosphatase | Xanthomonas axonopodis pv. citri str. 306 |
| 1jp4 | A | AMP | 3'(2'),5'-bisphosphate nucleotidase | Rattus norvegicus |
| 2uv4 | A | AMP | 5'-AMP-ACTIVATED PROTEIN KINASE SUBUNIT GAMMA-1 | Homo sapiens |
| 3e7w | A | AMP | D-alanine--poly(phosphoribitol) ligase subunit 1 | Bacillus subtilis |
| 2ptq | A | AMP | Adenylosuccinate lyase | Escherichia coli |
| 2hcr | A | AMP | Ribose-phosphate pyrophosphokinase I | Homo sapiens |
| 2i4i | A | AMP | ATP-dependent RNA helicase DDX3X | Homo sapiens |
| 1s68 | A | AMP | RNA Ligase 2 | Enterobacteria phage T4 |
| 3ddj | A | AMP | CBS domain-containing protein | Sulfolobus solfataricus |
| 2eqa | A | AMP | Hypothetical protein ST1526 | Sulfolobus tokodaii |
| 3lkm | A | AMP | Myosin heavy chain kinase A | Dictyostelium discoideum |
| 3fiu | A | AMP | NH(3)-dependent NAD(+) synthetase | Francisella tularensis subsp. holarctica LVS |
| 3ber | A | AMP | Probable ATP-dependent RNA helicase DDX47 | Homo sapiens |
| 2c5s | A | AMP | PROBABLE THIAMINE BIOSYNTHESIS PROTEIN THII | Bacillus anthracis str. Ames |
| 1h3d | A | AMP | ATP-PHOSPHORIBOSYLTRANSFERASE | Escherichia coli K-12 |
| 2jb7 | A | AMP | HYPOTHETICAL PROTEIN PAE2307 | Pyrobaculum aerophilum |
| 3i0q | A | AMP | Spectinomycin phosphotransferase | Legionella pneumophila serogroup 1 |
| 3lfr | A | AMP | putative Metal ion transporter | Pseudomonas syringae pv. tomato str. DC3000 |
| 2xxb | B | AMP | L-LACTATE DEHYDROGENASE | Thermus thermophilus HB8 |
| 1ry2 | A | AMP | acetyl-coenzyme A synthetase 1 | Saccharomyces cerevisiae |
| 1amu | A | AMP | GRAMICIDIN SYNTHETASE 1 | Brevibacillus brevis |
| 3err | B | AMP | fusion protein of microtubule binding domain from mouse cytoplasmic dynein and seryl-tRNA synthetase from Thermus thermophilus | Thermus thermophilus HB27 |
| 2ak3 | B | AMP | ADENYLATE KINASE ISOENZYME-3 | Bos taurus |
| 3l31 | A | AMP | Probable manganase-dependent inorganic pyrophosphatase | Clostridium perfringens str. 13 |
| 3qh8 | A | AMP | Beta-lactamase-like | Brucella melitensis biovar Abortus 2308 |
| 2gm3 | F | AMP | unknown protein | Arabidopsis thaliana |
| 1mc1 | A | AMP | BETA-LACTAM SYNTHETASE | Streptomyces clavuligerus |
| 3lud | C | AMP | Protein argonaute-2 | Homo sapiens |
| 1w0h | A | AMP | 3'-5' EXONUCLEASE ERI1 | Homo sapiens |
| 1mzv | A | AMP | Adenine Phosphoribosyltransferase | Leishmania tarentolae |
| 2hbl | A | AMP | Exosome complex exonuclease RRP6 | Saccharomyces cerevisiae |
| 3kd6 | B | AMP | Carbohydrate kinase, PfkB family | Chlorobaculum tepidum |
| 1ktg | A | AMP | Diadenosine Tetraphosphate Hydrolase | Caenorhabditis elegans |
| 3glv | B | AMP | Lipopolysaccharide core biosynthesis protein | Thermoplasma volcanium GSS1 |
| 1ecj | A | AMP | GLUTAMINE PHOSPHORIBOSYLPYROPHOSPHATE AMIDOTRANSFERASE | Escherichia coli |
| 2q4h | A | AMP | Probable galactose-1-phosphate uridyl transferase | Arabidopsis thaliana |
| 2x3k | A | AMP | ACSD | Erwinia chrysanthemi |
| 3g1z | A | AMP | Putative lysyl-tRNA synthetase | Salmonella enterica subsp. enterica serovar Typhimurium |
| 2qrk | A | AMP | Saccharopine dehydrogenase [NAD+, L-lysine-forming | Saccharomyces cerevisiae |
| 2dsd | B | AMP | ADP-sugar pyrophosphatase | Homo sapiens |
| 2rif | A | AMP | Conserved protein with 2 CBS domains | Pyrobaculum aerophilum str. IM2 |
| 1kpf | A | AMP | PROTEIN KINASE C INTERACTING PROTEIN | Homo sapiens |
| 3fwz | A | AMP | Inner membrane protein ybaL | Escherichia coli K-12 |
| 3nyo | A | AMP | G protein-coupled receptor kinase 6 | Homo sapiens |
| 3o0f | A | AMP | putative metal-dependent phosphoesterase | Bifidobacterium adolescentis ATCC 15703 |
| 2oun | A | AMP | cAMP and cAMP-inhibited cGMP 3',5'-cyclic phosphodiesterase 10A | Homo sapiens |
| 3m84 | A | AMP | Phosphoribosylformylglycinamidine cyclo-ligase | Francisella tularensis subsp. tularensis SCHU S4 |
| 3jwp | A | AMP | Transcriptional regulatory protein sir2 homologue | Plasmodium falciparum 3D7 |
| 2v8q | E | AMP | 5'-AMP-ACTIVATED PROTEIN KINASE CATALYTIC SUBUNIT ALPHA-1 | Rattus norvegicus |
| 1ua4 | A | AMP | ADP-dependent glucokinase | Pyrococcus furiosus |
| 3loq | B | AMP | universal stress protein | Archaeoglobus fulgidus |
| 2yb1 | A | AMP | AMIDOHYDROLASE | Chromobacterium violaceum |
| 3sbx | G | AMP | Putative uncharacterized protein | Mycobacterium marinum M |
| 1zn8 | A | AMP | Adenine phosphoribosyltransferase | Homo sapiens |
| 2a7x | A | AMP | Pantoate-beta-alanine ligase | Mycobacterium tuberculosis |
| 2vze | C | AMP | ACYL-COENZYME A SYNTHETASE ACSM2A, MITOCHONDRIAL | Homo sapiens |
| 3dlz | A | AMP | Serine/threonine-protein kinase haspin | Homo sapiens |
| 2qjt | A | AMP | Nicotinamide-nucleotide adenylyltransferase | Francisella tularensis |
| 3q10 | A | AMP | Pantoate--beta-alanine ligase | Yersinia pestis |
| 1t9g | S | AMP | Acyl-CoA dehydrogenase, medium-chain specific, mitochondrial | Homo sapiens |
| 2w4j | A | ADP | DEATH-ASSOCIATED PROTEIN KINASE 1 | Homo sapiens |
| 1th8 | A | ADP | Anti-sigma F factor | Geobacillus stearothermophilus |
| 3ihl | A | ADP | CTP synthase 2 | Homo sapiens |
| 2nun | A | ADP | Avirulence B protein | Pseudomonas syringae pv. glycinea |
| 1g41 | A | ADP | HEAT SHOCK PROTEIN HSLU | Haemophilus influenzae |
| 2wzb | A | ADP | PHOSPHOGLYCERATE KINASE 1 | Homo sapiens |
| 1vhl | A | ADP | Dephospho-CoA kinase | Escherichia coli |
| 3in1 | B | ADP | Uncharacterized sugar kinase ydjH | Escherichia coli K-12 |
| 3n83 | A | ADP | Aldehyde dehydrogenase, mitochondrial | Vulpes |
| 1y63 | A | ADP | Lmaj004144AAA protein | Leishmania major strain Friedlin |
| 2dya | A | ADP | Nucleoside diphosphate kinase | Pyrococcus horikoshii OT3 |
| 1o0h | A | ADP | Ribonuclease pancreatic | Bos taurus |
| 2hf3 | A | ADP | Actin-5C | Drosophila melanogaster |
| 3ocm | A | ADP | Putative membrane protein | Bordetella parapertussis |
| 2c98 | A | ADP | PSP OPERON TRANSCRIPTIONAL ACTIVATOR | Escherichia coli K-12 |
| 3kh5 | A | ADP | protein MJ1225 | Methanocaldococcus jannaschii |
| 2kin | A | ADP | KINESIN | Rattus norvegicus |
| 1fnn | A | ADP | CELL DIVISION CONTROL PROTEIN 6 | Pyrobaculum aerophilum |
| 1w2d | A | ADP | INOSITOL-TRISPHOSPHATE 3-KINASE A | Homo sapiens |
| 3kzb | A | ADP | Xylulokinase | Chromobacterium violaceum |
| 1zs6 | A | ADP | Nucleoside diphosphate kinase 3 | Homo sapiens |
| 1q3s | E | ADP | Thermosome alpha subunit | Thermococcus sp. KS-1 |
| 3a1d | A | ADP | Probable copper-exporting P-type ATPase A | Archaeoglobus fulgidus |
| 3auy | A | ADP | DNA double-strand break repair rad50 ATPase | Methanocaldococcus jannaschii |
| 2gry | A | ADP | Kinesin-like protein KIF2 | Homo sapiens |
| 1xx6 | A | ADP | Thymidine kinase | Clostridium acetobutylicum ATCC 824 |
| 2xd4 | A | ADP | PHOSPHORIBOSYLAMINE--GLYCINE LIGASE | Bacillus subtilis |
| 3lq3 | A | ADP | Choline/ethanolamine kinase | Homo sapiens |
| 2r87 | F | ADP | PurP protein PF1517 | Pyrococcus furiosus |
| 1nb0 | A | ADP | hypothetical protein FLJ11149 | Homo sapiens |
| 1iah | A | ADP | TRANSIENT RECEPTOR POTENTIAL-RELATED PROTEIN | Mus musculus |
| 1byq | A | ADP | PROTEIN (HEAT SHOCK PROTEIN 90) | Homo sapiens |
| 3cwq | A | ADP | ParA family chromosome partitioning protein | Synechocystis sp. PCC 6803 |
| 3ohr | A | ADP | Putative fructokinase | Bacillus subtilis |
| 2we5 | C | ADP | CARBAMATE KINASE 1 | Enterococcus faecalis |
| 3fyh | A | ADP | DNA repair and recombination protein radA | Methanococcus voltae |
| 2hru | A | ADP | Phosphoribosylformylglycinamidine synthase II | Thermotoga maritima |
| 2v1x | B | ADP | ATP-DEPENDENT DNA HELICASE Q1 | Homo sapiens |
| 2jls | A | ADP | SERINE PROTEASE SUBUNIT NS3 | Dengue virus 4 Thailand/0348/1991 |
| 1x3m | A | ADP | Propionate kinase | Salmonella enterica subsp. enterica serovar Typhimurium |
| 2pyw | B | ADP | Uncharacterized protein | Arabidopsis thaliana |
| 2ad5 | B | ADP | CTP synthase | Escherichia coli K-12 |
| 2if8 | A | ADP | Inositol polyphosphate multikinase | Saccharomyces cerevisiae |
| 2bej | A | ADP | SEGREGATION PROTEIN | Thermus thermophilus HB27 |
| 1h8e | C | ADP | BOVINE MITOCHONDRIAL F1-ATPASE | Bos taurus |
| 1n08 | B | ADP | putative riboflavin kinase | Schizosaccharomyces pombe |
| 2zdh | A | ADP | D-alanine--D-alanine ligase | Thermus thermophilus HB8 |
| 1dgk | N | ADP | HEXOKINASE TYPE I | Homo sapiens |
| 1rkd | A | ADP | RIBOKINASE | Escherichia coli K-12 |
| 2d7d | A | ADP | 5'-D(P*TP*TP*T)-3' | Bacillus subtilis |
| 3hbl | C | ADP | Pyruvate carboxylase | Staphylococcus aureus subsp. aureus Mu50 |
| 2bvc | B | ADP | GLUTAMINE SYNTHETASE 1 | Mycobacterium tuberculosis H37Rv |
| 2ncd | A | ADP | PROTEIN (Kinesin motor NCD) | Drosophila melanogaster |
| 2fna | A | ADP | Conserved hypothetical protein | Sulfolobus solfataricus P2 |
| 2v26 | A | ADP | MYOSIN VI | Sus scrofa |
| 1fwk | A | ADP | HOMOSERINE KINASE | Methanocaldococcus jannaschii |
| 2qzs | A | ADP | glycogen synthase | Escherichia coli |
| 1nkt | A | ADP | Preprotein translocase secA 1 subunit | Mycobacterium tuberculosis |
| 1k3d | A | ADP | Phosphoenolpyruvate carboxykinase | Escherichia coli |
| 2uyt | A | ADP | RHAMNULOKINASE | Escherichia coli |
| 3o8d | B | ADP | HCV NS3 protease/helicase | Hepatitis C virus subtype 1b |
| 2xzl | A | ADP | ATP-DEPENDENT HELICASE NAM7 | Saccharomyces cerevisiae |
| 2bkk | C | ADP | AMINOGLYCOSIDE 3'-PHOSPHOTRANSFERASE | Enterococcus faecalis |
| 3nua | A | ADP | Phosphoribosylaminoimidazole-succinocarboxamide synthase | Clostridium perfringens ATCC 13124 |
| 1r6b | X | ADP | ClpA protein | Escherichia coli |
| 1iqp | E | ADP | RFCS | Pyrococcus furiosus |
| 3pey | A | ADP | ATP-dependent RNA helicase DBP5 | Saccharomyces cerevisiae |
| 1lkx | D | ADP | MYOSIN IE HEAVY CHAIN | Dictyostelium discoideum |
| 1z2n | X | ADP | inositol 1,3,4-trisphosphate 5/6-kinase | Entamoeba histolytica HM-1:IMSS |
| 1g6o | A | ADP | CAG-ALPHA | Helicobacter pylori |
| 1eqm | A | ADP | 6-HYDROXYMETHYL-7,8-DIHYDROPTERIN PYROPHOSPHOKINASE | Escherichia coli |
| 1w5s | A | ADP | ORIGIN RECOGNITION COMPLEX SUBUNIT 2 ORC2 | Aeropyrum pernix |
| 2rio | B | ADP | Serine/threonine-protein kinase/endoribonuclease IRE1 | Saccharomyces cerevisiae |
| 2ch6 | D | ADP | N-ACETYL-D-GLUCOSAMINE KINASE | Homo sapiens |
| 3d8b | B | ADP | Fidgetin-like protein 1 | Homo sapiens |
| 1z6t | C | ADP | Apoptotic protease activating factor 1 | Homo sapiens |
| 2o8b | B | ADP | 5'-D(*GP*AP*AP*CP*CP*GP*CP*GP*CP*GP*CP*TP*AP*GP*G)-3' | Homo sapiens |
| 1v1a | B | ADP | 2-KETO-3-DEOXYGLUCONATE KINASE | Thermus thermophilus HB8 |
| 2zpa | A | ADP | Uncharacterized protein ypfI | Escherichia coli K-12 |
| 2oxc | A | ADP | Probable ATP-dependent RNA helicase DDX20 | Homo sapiens |
| 2obm | A | ADP | EscN | Escherichia coli O127:H6 str. E2348/69 |
| 1xw4 | X | ADP | Sulfiredoxin | Homo sapiens |
| 1svl | A | ADP | large T antigen | Simian virus 40 |
| 3i62 | A | ADP | ATP-dependent RNA helicase MSS116 | Saccharomyces cerevisiae |
| 1ohb | A | ADP | ACETYLGLUTAMATE KINASE | Escherichia coli |
| 1zth | A | ADP | Rio1 serine protein kinase | Archaeoglobus fulgidus |
| 1f9v | A | ADP | KINESIN-LIKE PROTEIN KAR3 | Saccharomyces cerevisiae |
| 3fi8 | A | ADP | Choline kinase | Plasmodium falciparum 3D7 |
| 2ako | A | ADP | Glutamate 5-kinase | Campylobacter jejuni |
| 3ncr | B | ADP | Nitrogen regulatory protein P-II (GlnB-2) | Archaeoglobus fulgidus |
| 1i5c | A | ADP | CHEMOTAXIS PROTEIN CHEA | Thermotoga maritima |
| 3r03 | A | ADP | NUDIX hydrolase | Rhodospirillum rubrum ATCC 11170 |
| 1u0j | A | ADP | DNA replication protein | Adeno-associated virus - 2 |
| 3kji | A | ADP | CO dehydrogenase/acetyl-CoA synthase complex, accessory protein CooC | Carboxydothermus hydrogenoformans Z-2901 |
| 2qv7 | A | ADP | Diacylglycerol Kinase DgkB | Staphylococcus aureus |
| 3f61 | A | ADP | Serine/threonine-protein kinase pknB | Mycobacterium tuberculosis |
| 3i73 | A | ADP | A-TYPE ATP SYNTHASE CATALYTIC SUBUNIT A | Pyrococcus horikoshii OT3 |
| 3a0t | A | ADP | Sensor protein | Thermotoga maritima |
| 1l8q | A | ADP | Chromosomal replication initiator protein dnaA | Aquifex aeolicus |
| 1m15 | A | ADP | arginine kinase | Limulus polyphemus |
| 2io9 | A | ADP | Bifunctional glutathionylspermidine synthetase/amidase | Escherichia coli |
| 3ice | A | ADP | Transcription termination factor rho | Escherichia coli K-12 |
| 3hfw | A | ADP | Protein ADP-ribosylarginine hydrolase | Homo sapiens |
| 2po0 | A | ADP | Probable exosome complex exonuclease 1 | Pyrococcus abyssi |
| 1o51 | A | ADP | Hypothetical protein TM0021 | Thermotoga maritima |
| 1um8 | A | ADP | ATP-dependent Clp protease ATP-binding subunit clpX | Helicobacter pylori 26695 |
| 1e8h | A | ADP | VANILLYL-ALCOHOL OXIDASE | Penicillium simplicissimum |
| 2ww4 | A | ADP | 4-DIPHOSPHOCYTIDYL-2C-METHYL-D-ERYTHRITOL KINASE | Escherichia coli |
| 1zar | A | ADP | Rio2 kinase | Archaeoglobus fulgidus |
| 2hv7 | E | ADP | Protein phosphatase 2A, regulatory subunit B | Homo sapiens |
| 1uky | A | ADP | URIDYLATE KINASE | Saccharomyces cerevisiae |
| 2iyv | A | ADP | SHIKIMATE KINASE | Mycobacterium tuberculosis H37Rv |
| 3a4m | A | ADP | L-seryl-tRNA(Sec) kinase | Methanocaldococcus jannaschii |
| 3fmo | B | ADP | Nuclear pore complex protein Nup214 | Homo sapiens |
| 1dad | A | ADP | DETHIOBIOTIN SYNTHETASE | Escherichia coli |
| 2ax4 | A | ADP | Bifunctional 3'-phosphoadenosine 5'-phosphosulfate synthetase 2 | Homo sapiens |
| 3ko5 | A | ADP | D-tyrosyl-tRNA(Tyr) deacylase | Plasmodium falciparum 3D7 |
| 1x6v | B | ADP | Bifunctional 3'-phosphoadenosine 5'-phosphosulfate synthetase 1 | Homo sapiens |
| 2xzo | A | ADP | REGULATOR OF NONSENSE TRANSCRIPTS 1 | Homo sapiens |
| 3pdt | A | ADP | Myosin heavy chain kinase A | Dictyostelium discoideum |
| 3kb1 | B | ADP | Nucleotide-binding protein | Archaeoglobus fulgidus |
| 3i33 | A | ADP | Heat shock-related 70 kDa protein 2 | Homo sapiens |
| 2d0o | A | ADP | diol dehydratase-reactivating factor large subunit | Klebsiella oxytoca |
| 2c2a | A | ADP | SENSOR HISTIDINE KINASE | Thermotoga maritima |
| 2v7y | A | ADP | CHAPERONE PROTEIN DNAK | Geobacillus kaustophilus HTA426 |
| 3bf1 | C | ADP | Type III pantothenate kinase | Thermotoga maritima |
| 3fh0 | A | ADP | putative universal stress protein KPN_01444 | Klebsiella pneumoniae subsp. pneumoniae MGH 78578 |
| 1ihu | A | ADP | ARSENICAL PUMP-DRIVING ATPASE | Escherichia coli |
| 3p23 | C | ADP | Serine/threonine-protein kinase/endoribonuclease IRE1 | Homo sapiens |
| 1g3q | A | ADP | CELL DIVISION INHIBITOR | Pyrococcus furiosus |
| 1b6s | B | ADP | PROTEIN (N5-CARBOXYAMINOIMIDAZOLE RIBONUCLEOTIDE SYNTHETASE) | Escherichia coli |
| 1nva | B | ADP | 3-DEHYDROQUINATE SYNTHASE | Emericella nidulans |
| 2ojw | B | ADP | Glutamine synthetase | Homo sapiens |
| 1htw | A | ADP | HI0065 | Haemophilus influenzae |
| 3n2a | A | ADP | Bifunctional folylpolyglutamate synthase/dihydrofolate synthase | Yersinia pestis CO92 |
| 3fwr | A | ADP | YqzB protein | Bacillus subtilis |
| 3llm | A | ADP | ATP-dependent RNA helicase A | Homo sapiens |
| 3fwy | A | ADP | Light-independent protochlorophyllide reductase iron-sulfur ATP-binding protein | Rhodobacter sphaeroides 2.4.1 |
| 2bfr | A | ADP | HYPOTHETICAL PROTEIN AF1521 | Archaeoglobus fulgidus |
| 3rv3 | B | ADP | Biotin carboxylase | Escherichia coli K-12 |
| 3jv2 | A | ADP | Protein translocase subunit secA | Bacillus subtilis |
| 3ec2 | A | ADP | DNA replication protein DnaC | Aquifex aeolicus |
| 1t3t | A | ADP | Phosphoribosylformylglycinamidine synthase | Salmonella enterica subsp. enterica serovar Typhimurium |
| 1b62 | A | ADP | PROTEIN (MUTL) | Escherichia coli K-12 |
| 2iuu | C | ADP | DNA TRANSLOCASE FTSK | Pseudomonas aeruginosa |
| 3bxz | B | ADP | Preprotein translocase subunit secA | Escherichia coli |
| 2zj5 | A | ADP | Putative ski2-type helicase | Pyrococcus furiosus |
| 3l8k | A | ADP | Dihydrolipoyl dehydrogenase | Sulfolobus solfataricus |
| 3fd6 | A | ADP | Selenide, water dikinase 1 | Homo sapiens |
| 1ltq | A | ADP | POLYNUCLEOTIDE KINASE | Enterobacteria phage T4 |
| 3akk | A | ADP | CtkA | Helicobacter pylori J99 |
| 2w41 | A | ADP | GLYCEROL KINASE, PUTATIVE | Plasmodium falciparum 3D7 |
| 1u3f | A | ADP | 5,10-Methenyltetrahydrofolate Synthetase | Mycoplasma pneumoniae |
| 1rfv | A | ADP | pyridoxal kinase | Ovis aries |
| 1mwm | B | ADP | ParM | Escherichia coli |
| 1ny5 | A | ADP | transcriptional regulator (NtrC family) | Aquifex aeolicus |
| 3iij | A | ADP | Coilin-interacting nuclear ATPase protein | Homo sapiens |
| 1fp6 | D | ADP | NITROGENASE IRON PROTEIN | Azotobacter vinelandii |
| 2yx6 | C | ADP | Hypothetical protein PH0822 | Pyrococcus horikoshii OT3 |
| 3gvi | E | ADP | Malate dehydrogenase | Brucella melitensis biovar Abortus 2308 |
| 3hyo | A | ADP | Pyridoxal kinase | Lactobacillus plantarum |
| 3d2r | A | ADP | [Pyruvate dehydrogenase [lipoamide]] kinase isozyme 4 | Homo sapiens |
| 3lrt | A | ADP | Ribose-phosphate pyrophosphokinase | Thermoplasma volcanium |
| 1sq5 | C | ADP | Pantothenate kinase | Escherichia coli |
| 2z4s | A | ADP | Chromosomal replication initiator protein dnaA | Thermotoga maritima |
| 1s3s | B | ADP | Transitional endoplasmic reticulum ATPase (TER ATPase) (15S Mg(2+)- ATPase p97 subunit) (Valosin containing protein) (VCP) [Contains: Valosin] | Mus musculus |
| 3g2f | B | ADP | Bone morphogenetic protein receptor type-2 | Homo sapiens |
| 3kqn | A | ADP | Serine protease/NTPase/helicase NS3 | Hepatitis C virus (isolate Con1) |
| 2dpy | B | ADP | Flagellum-specific ATP synthase | Salmonella enterica subsp. enterica serovar Typhimurium |
| 2zts | C | ADP | Putative uncharacterized protein PH0186 | Pyrococcus horikoshii |
| 2q14 | B | ADP | Phosphohydrolase | Bacteroides thetaiotaomicron VPI-5482 |
| 2uvq | A | ADP | URIDINE-CYTIDINE KINASE 1 | Homo sapiens |
| 1in4 | A | ADP | HOLLIDAY JUNCTION DNA HELICASE RUVB | Thermotoga maritima |
| 3ez2 | A | ADP | Plasmid partition protein A | Escherichia coli |
| 1zxn | B | ADP | DNA topoisomerase II, alpha isozyme | Homo sapiens |
| 3ehh | A | ADP | Sensor kinase (YocF protein) | Bacillus subtilis |
| 3cr3 | A | ADP | PTS-dependent dihydroxyacetone kinase, ADP-binding subunit dhaL | Lactococcus lactis subsp. lactis Il1403 |
| 1gc5 | A | ADP | ADP-DEPENDENT GLUCOKINASE | Thermococcus litoralis |
| 3qbw | B | ADP | Anhydro-N-acetylmuramic acid kinase | Pseudomonas aeruginosa |
| 3ll9 | A | ADP | Isopentenyl phosphate kinase | Methanothermobacter thermautotrophicus |
| 1t6x | B | ADP | riboflavin kinase/FMN adenylyltransferase | Thermotoga maritima |
| 3qc9 | D | ADP | Rhodopsin kinase | Bos taurus |
| 3egi | B | ADP | Trimethylguanosine synthase homolog | Homo sapiens |
| 3dkp | A | ADP | Probable ATP-dependent RNA helicase DDX52 | Homo sapiens |
| 1wnl | A | ADP | biotin--[acetyl-CoA-carboxylase] ligase | Pyrococcus horikoshii OT3 |
| 1gkz | A | ADP | [3-METHYL-2-OXOBUTANOATE DEHYDROGENASE [LIPOAMIDE]] KINASE | Rattus norvegicus |
| 2ce7 | A | ADP | CELL DIVISION PROTEIN FTSH | Thermotoga maritima |
| 1z5b | A | ADP | Type II DNA topoisomerase VI subunit B | Sulfolobus shibatae |
| 3k0s | A | ADP | DNA mismatch repair protein mutS | Escherichia coli K-12 |
| 2vhj | B | ADP | NTPASE P4 | Pseudomonas phage phi12 |
| 2zro | A | ADP | Protein recA | Mycobacterium smegmatis str. MC2 155 |
| 1p5z | B | ADP | Deoxycytidine kinase | Homo sapiens |
| 3bfv | B | ADP | Membrane protein CapA1, Protein tyrosine kinase | Staphylococcus aureus |
| 2h1f | B | ADP | Lipopolysaccharide heptosyltransferase-1 | Escherichia coli O6 |
| 3fjq | E | ATP | cAMP-dependent protein kinase catalytic subunit alpha | Mus musculus |
| 1jjv | A | ATP | DEPHOSPHO-COA KINASE | Haemophilus influenzae |
| 2pnn | A | ATP | Transient receptor potential cation channel subfamily V member 1 | Rattus norvegicus |
| 1i7l | B | ATP | SYNAPSIN II | Rattus norvegicus |
| 1do0 | D | ATP | PROTEIN (HEAT SHOCK LOCUS U) | Escherichia coli |
| 1vjd | A | ATP | phosphoglycerate kinase | Sus scrofa |
| 1g5t | A | ATP | COB(I)AL | Salmonella enterica subsp. enterica serovar Typhimurium |
| 3rgl | A | ATP | Glycyl-tRNA synthetase alpha subunit | Campylobacter jejuni |
| 2j9l | E | ATP | CHLORIDE CHANNEL PROTEIN 5 | Homo sapiens |
| 2w5i | A | ATP | RIBONUCLEASE PANCREATIC | Bos taurus |
| 1qz5 | A | ATP | Actin, alpha skeletal muscle | Oryctolagus cuniculus |
| 1ko5 | B | ATP | Gluconate kinase | Escherichia coli |
| 1sx3 | B | ATP | groEL protein | Escherichia coli |
| 1ojl | E | ATP | TRANSCRIPTIONAL REGULATORY PROTEIN ZRAR | Salmonella enterica subsp. enterica serovar Typhimurium |
| 2zan | A | ATP | Vacuolar protein sorting-associating protein 4B | Mus musculus |
| 3lfz | A | ATP | protein MJ1225 | Methanocaldococcus jannaschii |
| 3ea0 | A | ATP | ATPase, ParA family | Chlorobium tepidum TLS |
| 2z08 | A | ATP | Universal stress protein family | Thermus thermophilus |
| 2ogx | A | ATP | Molybdenum storage protein subunit alpha | Azotobacter vinelandii |
| 2yw2 | A | ATP | Phosphoribosylamine--glycine ligase | Aquifex aeolicus |
| 3i7v | A | ATP | AP4A hydrolase | Aquifex aeolicus VF5 |
| 1nsf | A | ATP | N-ETHYLMALEIMIDE SENSITIVE FACTOR | Cricetulus griseus |
| 2r86 | A | ATP | PurP protein PF1517 | Pyrococcus furiosus |
| 3gni | B | ATP | Protein Mo25 | Homo sapiens |
| 1xex | A | ATP | SMC protein | Pyrococcus furiosus |
| 3h1q | A | ATP | Ethanolamine utilization protein EutJ | Carboxydothermus hydrogenoformans Z-2901 |
| 1yun | A | ATP | Probable nicotinate-nucleotide adenylyltransferase | Pseudomonas aeruginosa |
| 2zt7 | A | ATP | Glycyl-tRNA synthetase | Homo sapiens |
| 3rk1 | A | ATP | N-type ATP pyrophosphatase superfamily | Pyrococcus furiosus DSM 3638 |
| 2o0h | A | ATP | DNA packaging protein Gp17 | Enterobacteria phage T4 |
| 2w00 | A | ATP | HSDR | Escherichia coli |
| 2v92 | E | ATP | 5'-AMP-ACTIVATED PROTEIN KINASE CATALYTIC SUBUNIT ALPHA-1 | Rattus norvegicus |
| 2qkm | F | ATP | SPBC3B9.21 protein | Schizosaccharomyces pombe |
| 1yfr | B | ATP | Alanyl-tRNA synthetase | Aquifex aeolicus |
| 2fgh | A | ATP | gelsolin | Equus caballus |
| 1n75 | A | ATP | Glutamyl-tRNA synthetase | Thermus thermophilus |
| 2hs0 | A | ATP | Phosphoribosylformylglycinamidine synthase II | Thermotoga maritima |
| 2y27 | A | ATP | PHENYLACETATE-COENZYME A LIGASE | Burkholderia cenocepacia J2315 |
| 2bek | A | ATP | SEGREGATION PROTEIN | Thermus thermophilus HB27 |
| 2r9v | A | ATP | ATP synthase subunit alpha | Thermotoga maritima MSB8 |
| 3r5f | A | ATP | D-alanine--D-alanine ligase 1 | Xanthomonas oryzae pv. oryzae KACC 10331 |
| 3ikh | C | ATP | Carbohydrate kinase | Klebsiella pneumoniae subsp. pneumoniae MGH 78578 |
| 2q0d | A | ATP | RNA uridylyl transferase | Trypanosoma brucei |
| 1y8q | D | ATP | Ubiquitin-like 1 activating enzyme E1A | Homo sapiens |
| 3f5m | B | ATP | 6-phospho-1-fructokinase (ATP-dependent phosphofructokinase) | Trypanosoma brucei |
| 1fmw | A | ATP | MYOSIN II HEAVY CHAIN | Dictyostelium discoideum |
| 2ddo | A | ATP | Pyridoxine kinase | Escherichia coli |
| 3fcc | A | ATP | D-alanine--poly(phosphoribitol) ligase subunit 1 | Bacillus cereus ATCC 14579 |
| 1esq | A | ATP | HYDROXYETHYLTHIAZOLE KINASE | Bacillus subtilis |
| 2olr | A | ATP | Phosphoenolpyruvate carboxykinase | Escherichia coli K-12 |
| 1qhx | A | ATP | PROTEIN (CHLORAMPHENICOL PHOSPHOTRANSFERASE) | Streptomyces venezuelae |
| 1csn | A | ATP | CASEIN KINASE-1 | Schizosaccharomyces pombe |
| 1r8b | A | ATP | tRNA nucleotidyltransferase | Archaeoglobus fulgidus |
| 3lmi | A | ATP | Myosin heavy chain kinase A | Dictyostelium discoideum |
| 3r5x | B | ATP | D-alanine--D-alanine ligase | Bacillus anthracis |
| 3qxc | A | ATP | Dethiobiotin synthetase | Helicobacter pylori 26695 |
| 1f9a | F | ATP | HYPOTHETICAL PROTEIN MJ0541 | Methanocaldococcus jannaschii |
| 3nd6 | C | ATP | Phosphopantetheine adenylyltransferase | Enterococcus faecalis |
| 2aru | A | ATP | Lipoate-protein ligase A | Thermoplasma acidophilum |
| 1xdn | A | ATP | RNA editing ligase MP52 | Trypanosoma brucei |
| 3iq0 | A | ATP | putative Ribokinase II | Escherichia coli O6 |
| 3gqk | A | ATP | Preneck appendage protein | Bacillus phage phi29 |
| 3hy2 | X | ATP | Peroxiredoxin-1 | Homo sapiens |
| 3bju | A | ATP | Lysyl-tRNA synthetase | Homo sapiens |
| 1svm | D | ATP | large T antigen | Simian virus 40 |
| 1zp9 | B | ATP | Rio1 kinase | Archaeoglobus fulgidus DSM 4304 |
| 2e5y | B | ATP | ATP synthase epsilon chain | Bacillus sp. PS3 |
| 2xbp | A | ATP | NITROGEN REGULATORY PROTEIN P-II | Synechococcus elongatus PCC 7942 |
| 2c96 | A | ATP | PSP OPERON TRANSCRIPTIONAL ACTIVATOR | Escherichia coli K-12 |
| 3ab8 | A | ATP | Putative uncharacterized protein TTHA0350 | Thermus thermophilus HB8 |
| 3r78 | A | ATP | Aminoglycoside 3'-phosphotransferase AphA1-IAB | Acinetobacter baumannii AYE |
| 3bg5 | C | ATP | Pyruvate carboxylase | Staphylococcus aureus |
| 3gah | A | ATP | Cobalamin adenosyltransferase PduO-like protein | Lactobacillus reuteri |
| 2yww | A | ATP | Aspartate carbamoyltransferase regulatory chain | Methanocaldococcus jannaschii |
| 3lss | B | ATP | Seryl-tRNA synthetase | Trypanosoma brucei |
| 2yxu | B | ATP | Pyridoxal kinase | Homo sapiens |
| 1yp3 | C | ATP | Glucose-1-phosphate adenylyltransferase small subunit | Solanum tuberosum |
| 3s3t | A | ATP | Nucleotide-binding protein, universal stress protein UspA family | Lactobacillus plantarum |
| 3h5n | D | ATP | MccB protein | Escherichia coli |
| 1f2u | C | ATP | RAD50 ABC-ATPASE | Pyrococcus furiosus |
| 2q66 | A | ATP | 5'-R(P*AP*AP*AP*AP*A)-3' | Saccharomyces cerevisiae |
| 1zao | A | ATP | Rio2 serine kinase | Archaeoglobus fulgidus |
| 3rep | A | ATP | Integrin-linked kinase | Homo sapiens |
| 2iyw | A | ATP | SHIKIMATE KINASE | Mycobacterium tuberculosis H37Rv |
| 3am1 | A | ATP | L-seryl-tRNA(Sec) kinase | Methanocaldococcus jannaschii |
| 1zfn | C | ATP | Adenylyltransferase thiF | Escherichia coli |
| 1xdp | A | ATP | Polyphosphate kinase | Escherichia coli |
| 1kp2 | A | ATP | argininosuccinate synthetase | Escherichia coli |
| 2vt3 | A | ATP | REDOX-SENSING TRANSCRIPTIONAL REPRESSOR REX | Bacillus subtilis |
| 2hix | A | ATP | Thermostable DNA ligase | Sulfolobus solfataricus |
| 3lkk | B | ATP | Gamma-glutamyl kinase related protein | Thermoplasma acidophilum DSM 1728 |
| 1mb9 | B | ATP | BETA-LACTAM SYNTHETASE | Streptomyces clavuligerus |
| 3jzm | A | ATP | Circadian clock protein kinase kaiC | Synechococcus elongatus PCC 7942 |
| 3qx9 | A | ATP | Protein argonaute-2 | Homo sapiens |
| 1mjh | A | ATP | PROTEIN (ATP-BINDING DOMAIN OF PROTEIN MJ0577) | Methanocaldococcus jannaschii |
| 1kax | A | ATP | 70KD HEAT SHOCK COGNATE PROTEIN | Bos taurus |
| 3fdx | A | ATP | Putative filament protein / universal stress protein F | Klebsiella pneumoniae subsp. pneumoniae MGH 78578 |
| 3h8v | A | ATP | Ubiquitin-like modifier-activating enzyme 5 | Homo sapiens |
| 3eps | B | ATP | Isocitrate dehydrogenase kinase/phosphatase | Escherichia coli O157:H7 |
| 2a5y | B | ATP | Apoptosis regulator ced-9 | Caenorhabditis elegans |
| 1pk8 | A | ATP | rat synapsin I | Rattus norvegicus |
| 1hp1 | A | ATP | 5'-NUCLEOTIDASE | Escherichia coli |
| 2jjx | A | ATP | URIDYLATE KINASE | Bacillus anthracis str. Ames |
| 1su2 | B | ATP | MutT/nudix family protein | Deinococcus radiodurans |
| 3e7e | A | ATP | Mitotic checkpoint serine/threonine-protein kinase BUB1 | Homo sapiens |
| 1dv2 | A | ATP | BIOTIN CARBOXYLASE | Escherichia coli |
| 3na3 | A | ATP | DNA mismatch repair protein Mlh1 | Homo sapiens |
| 2w02 | B | ATP | ACHROMOBACTIN SYNTHETASE PROTEIN D | Erwinia chrysanthemi |
| 3lki | B | ATP | Fructokinase | Xylella fastidiosa Temecula1 |
| 1b8a | A | ATP | PROTEIN (ASPARTYL-TRNA SYNTHETASE) | Thermococcus kodakarensis KOD1 |
| 2npi | B | ATP | Protein CLP1 | Saccharomyces cerevisiae |
| 2e89 | B | ATP | tRNA(Ile)-lysidine synthase | Aquifex aeolicus |
| 3gbu | D | ATP | Uncharacterized sugar kinase PH1459 | Pyrococcus horikoshii OT3 |
| 2nvu | B | ATP | NEDD8-activating enzyme E1 regulatory subunit | Homo sapiens |
| 2c8v | A | ATP | NITROGENASE IRON PROTEIN 1 | Azotobacter vinelandii |
| 2w74 | D | ATP | TYPE I RESTRICTION ENZYME ECOR124II R PROTEIN | Escherichia coli |
| 3ibq | A | ATP | Pyridoxal kinase | Lactobacillus plantarum |
| 1j21 | A | ATP | Argininosuccinate Synthetase | Thermus thermophilus |
| 1a0i | A | ATP | DNA LIGASE | Enterobacteria phage T7 |
| 1z0s | A | ATP | Probable inorganic polyphosphate/ATP-NAD kinase | Archaeoglobus fulgidus |
| 3eth | A | ATP | Phosphoribosylaminoimidazole carboxylase ATPase subunit | Escherichia coli K-12 |
| 3h39 | A | ATP | TRNA nucleotidyl transferase-related protein | Thermotoga maritima |
| 1miw | A | ATP | tRNA CCA-adding enzyme | Geobacillus stearothermophilus |
| 3d2f | A | ATP | Heat shock protein homolog SSE1 | Saccharomyces cerevisiae |
| 1kvk | A | ATP | mevalonate kinase | Rattus norvegicus |
| 3dnt | A | ATP | Protein hipA | Escherichia coli K-12 |
| 1j7k | A | ATP | HOLLIDAY JUNCTION DNA HELICASE RUVB | Thermotoga maritima |
| 3m0e | C | ATP | Transcriptional regulator (NtrC family) | Aquifex aeolicus |
| 2x0q | A | ATP | ALCALIGIN BIOSYNTHESIS PROTEIN | Bordetella bronchiseptica |
| 3ehg | A | ATP | Sensor kinase (YocF protein) | Bacillus subtilis |
| 2a84 | A | ATP | Pantoate--beta-alanine ligase | Mycobacterium tuberculosis |
| 3efs | A | ATP | Biotin [acetyl-CoA-carboxylase] ligase | Aquifex aeolicus |
| 1e8x | A | ATP | PHOSPHATIDYLINOSITOL 3-KINASE CATALYTIC SUBUNIT | Sus scrofa |
| 1w7a | B | ATP | DNA MISMATCH REPAIR PROTEIN MUTS | Escherichia coli |
| 2vhq | B | ATP | NTPASE P4 | Pseudomonas phage phi12 |
| 1zyd | A | ATP | Serine/threonine-protein kinase GCN2 | Saccharomyces cerevisiae |
| 2ivp | A | ATP | O-SIALOGLYCOPROTEIN ENDOPEPTIDASE | Pyrococcus abyssi |
| 3ie7 | A | ATP | Lin2199 protein | Listeria innocua |
| 3g59 | A | ATP | FMN Adenylyltransferase | Candida glabrata |
| 3nsz | A | ANP | Casein kinase II subunit alpha | Homo sapiens |
| 1vpe | A | ANP | PHOSPHOGLYCERATE KINASE | Thermotoga maritima |
| 3d41 | A | ANP | FomA protein | Streptomyces wedmorensis |
| 2p9u | A | ANP | Actin-like protein 3 | Bos taurus |
| 2c99 | A | ANP | PSP OPERON TRANSCRIPTIONAL ACTIVATOR | Escherichia coli K-12 |
| 1q3q | A | ANP | Thermosome alpha subunit | Thermococcus sp. KS-1 |
| 3qf7 | A | ANP | Rad50 | Thermotoga maritima |
| 1v8k | A | ANP | Kinesin-like protein KIF2C | Mus musculus |
| 2xcl | A | ANP | PHOSPHORIBOSYLAMINE--GLYCINE LIGASE | Bacillus subtilis |
| 1d2n | A | ANP | N-ETHYLMALEIMIDE-SENSITIVE FUSION PROTEIN | Cricetulus griseus |
| 1ia9 | A | ANP | TRANSIENT RECEPTOR POTENTIAL-RELATED PROTEIN | Mus musculus |
| 2yef | A | ANP | HEAT SHOCK PROTEIN HSP 90-ALPHA | Homo sapiens |
| 2wtk | B | ANP | CALCIUM-BINDING PROTEIN 39 | Homo sapiens |
| 1zy5 | A | ANP | Serine/threonine-protein kinase GCN2 | Saccharomyces cerevisiae |
| 2jlr | A | ANP | SERINE PROTEASE SUBUNIT NS3 | Dengue virus 4 Thailand/0348/1991 |
| 1x3n | A | ANP | Propionate kinase | Salmonella enterica subsp. enterica serovar Typhimurium |
| 2o1u | A | ANP | Endoplasmin | Canis lupus familiaris |
| 1m0w | A | ANP | glutathione synthetase | Saccharomyces cerevisiae |
| 1e1q | F | ANP | BOVINE MITOCHONDRIAL F1-ATPASE | Bos taurus |
| 3n8d | A | ANP | D-alanine--D-alanine ligase | Staphylococcus aureus |
| 3aar | A | ANP | Ectonucleoside triphosphate diphosphohydrolase I | Legionella pneumophila subsp. pneumophila str. Philadelphia 1 |
| 1id0 | A | ANP | PHOQ HISTIDINE KINASE | Escherichia coli |
| 3ih0 | A | ANP | Uncharacterized sugar kinase PH1459 | Pyrococcus horikoshii |
| 2pml | X | ANP | Ser/Thr protein kinase | Plasmodium falciparum |
| 1mmn | A | ANP | MYOSIN | Dictyostelium discoideum |
| 3guc | A | ANP | Ubiquitin-like modifier-activating enzyme 5 | Homo sapiens |
| 1j7u | B | ANP | AMINOGLYCOSIDE 3'-PHOSPHOTRANSFERASE | Enterococcus faecalis |
| 3fht | A | ANP | ATP-dependent RNA helicase DDX19B | Homo sapiens |
| 3aje | A | ANP | Putative uncharacterized protein ST1526 | Sulfolobus tokodaii str. 7 |
| 3kn5 | A | ANP | Ribosomal protein S6 kinase alpha-5 | Homo sapiens |
| 1jnk | A | ANP | C-JUN N-TERMINAL KINASE | Homo sapiens |
| 2i1q | A | ANP | DNA repair and recombination protein radA | Methanococcus voltae |
| 3b7g | A | ANP | Probable ATP-dependent RNA helicase DDX20 | Homo sapiens |
| 3gpl | A | ANP | Exodeoxyribonuclease V, subunit RecD, putative | Deinococcus radiodurans R1 |
| 3i5x | A | ANP | ATP-dependent RNA helicase MSS116 | Saccharomyces cerevisiae |
| 1gs5 | A | ANP | ACETYLGLUTAMATE KINASE | Escherichia coli |
| 1vfv | A | ANP | PROTEIN (Fusion protein consisting of Kinesin-like protein KIF1A, Kinesin heavy chain isoform 5C and A HIS TAG | Mus musculus |
| 2p0a | A | ANP | Synapsin-3 | Homo sapiens |
| 1i5b | B | ANP | CHEMOTAXIS PROTEIN CHEA | Thermotoga maritima |
| 2q7d | B | ANP | Inositol-tetrakisphosphate 1-kinase | Homo sapiens |
| 3ju6 | A | ANP | Arginine kinase | Apostichopus japonicus |
| 3lij | A | ANP | Calcium/calmodulin dependent protein kinase with a kinase domain and 4 calmodulin like EF hands | Cryptosporidium parvum Iowa II |
| 2ajp | A | ANP | Pyridoxal kinase | Homo sapiens |
| 1wuu | B | ANP | Galactokinase | Homo sapiens |
| 2aj4 | A | ANP | Galactokinase | Saccharomyces cerevisiae |
| 2qoc | A | ANP | Ephrin receptor | Homo sapiens |
| 2q7e | A | ANP | Pyrrolysyl-tRNA synthetase | Methanosarcina mazei |
| 1tqm | A | ANP | conserved hypothetical protein | Archaeoglobus fulgidus DSM 4304 |
| 3ng0 | A | ANP | Glutamine synthetase | Synechocystis sp. PCC 6803 |
| 3baf | A | ANP | Shikimate kinase | Mycobacterium tuberculosis |
| 3add | B | ANP | L-seryl-tRNA(Sec) kinase | Methanocaldococcus jannaschii DSM 2661 |
| 3qxs | A | ANP | Dethiobiotin synthetase | Helicobacter pylori 26695 |
| 2gjk | A | ANP | Regulator of nonsense transcripts 1 | Homo sapiens |
| 2e8a | A | ANP | Heat shock 70kDa protein 1A | Homo sapiens |
| 2db3 | C | ANP | 5'-R(*UP*UP*UP*UP*UP*UP*UP*UP*UP*U)-3' | Drosophila melanogaster |
| 1pvg | B | ANP | DNA topoisomerase II | Saccharomyces cerevisiae |
| 2bri | A | ANP | URIDYLATE KINASE | Pyrococcus furiosus |
| 3pyz | A | ANP | Bifunctional folylpolyglutamate synthase/dihydrofolate synthase | Yersinia pestis CO92 |
| 3fws | B | ANP | YqzB protein | Bacillus subtilis |
| 3qkt | A | ANP | DNA double-strand break repair rad50 ATPase | Pyrococcus furiosus |
| 2a2d | A | ANP | N-acetylgalactosamine kinase | Homo sapiens |
| 1b63 | A | ANP | MUTL | Escherichia coli K-12 |
| 2src | A | ANP | TYROSINE-PROTEIN KINASE SRC | Homo sapiens |
| 3fgu | A | ANP | Glucokinase | Homo sapiens |
| 2hw1 | A | ANP | Ketohexokinase | Homo sapiens |
| 3akl | A | ANP | Ctka | Helicobacter pylori J99 |
| 1e0j | E | ANP | DNA HELICASE | Enterobacteria phage T7 |
| 2vug | A | ANP | PAB1020 | Pyrococcus abyssi GE5 |
| 2e21 | A | ANP | tRNA(Ile)-lysidine synthase | Aquifex aeolicus |
| 2mjp | A | ANP | PYROPHOSPHATASE | Methanocaldococcus jannaschii |
| 2e0a | A | ANP | Pyruvate dehydrogenase kinase isozyme 4 | Homo sapiens |
| 1esn | A | ANP | PANTOTHENATE KINASE | Escherichia coli |
| 1qvr | A | ANP | ClpB protein | Thermus thermophilus |
| 1s16 | B | ANP | Topoisomerase IV subunit B | Escherichia coli |
| 2zue | A | ANP | Arginyl-tRNA synthetase | Pyrococcus horikoshii |
| 2ozo | A | ANP | Tyrosine-protein kinase ZAP-70 | Homo sapiens |
| 1v25 | B | ANP | long-chain-fatty-acid-CoA synthetase | Thermus thermophilus |
| 1mx0 | C | ANP | Type II DNA topoisomerase VI subunit B | Sulfolobus shibatae |
| 1q99 | A | ANP | SR protein kinsae | Saccharomyces cerevisiae |
| 1xms | A | ANP | RecA protein | Escherichia coli |
| 2zgy | A | GDP | Plasmid segregation protein parM | Escherichia coli |
| 1tad | A | GDP | TRANSDUCIN-ALPHA | Bos taurus |
| 2qnr | A | GDP | Septin-2 | Homo sapiens |
| 1rq7 | A | GDP | Cell division protein ftsZ | Mycobacterium tuberculosis |
| 3af1 | A | GDP | Pantothenate kinase | Mycobacterium tuberculosis H37Rv |
| 3ieu | A | GDP | GTP-binding protein era | Escherichia coli K-12 |
| 3kkq | A | GDP | Ras-related protein M-Ras | Mus musculus |
| 2ywh | A | GDP | GTP-binding protein LepA | Aquifex aeolicus VF5 |
| 2phn | B | GDP | F420-0:gamma-glutamyl ligase | Archaeoglobus fulgidus DSM 4304 |
| 3o47 | B | GDP | ADP-ribosylation factor GTPase-activating protein 1, ADP-ribosylation factor 1 | Homo sapiens |
| 3a1s | A | GDP | Iron(II) transport protein B | Thermotoga maritima |
| 2ng1 | A | GDP | SIGNAL SEQUENCE RECOGNITION PROTEIN FFH | Thermus aquaticus |
| 3dtb | A | GDP | Phosphoenolpyruvate carboxykinase, cytosolic [GTP] | Rattus norvegicus |
| 3dm5 | A | GDP | Signal recognition 54 kDa protein | Pyrococcus furiosus |
| 2dxe | A | GDP | Nucleoside diphosphate kinase | Pyrococcus horikoshii OT3 |
| 3cb2 | B | GDP | tubulin gamma-1 chain | Homo sapiens |
| 2rcn | A | GDP | Probable GTPase engC | Salmonella enterica subsp. enterica serovar Typhimurium str. LT2 |
| 3pqc | A | GDP | Probable GTP-binding protein engB | Thermotoga maritima |
| 3def | A | GDP | T7I23.11 protein | Arabidopsis thaliana |
| 2xkb | A | GDP | FTSZ/TUBULIN-RELATED PROTEIN | Bacillus thuringiensis serovar israelensis |
| 1tq4 | A | GDP | interferon-inducible GTPase | Mus musculus |
| 1mky | A | GDP | Probable GTP-binding protein engA | Thermotoga maritima |
| 1zny | A | GDP | Guanylate kinase | Mycobacterium tuberculosis |
| 2qm7 | A | GDP | GTPase/ATPase | Methylobacterium extorquens AM1 |
| 1rya | A | GDP | GDP-mannose mannosyl hydrolase | Escherichia coli |
| 1kk3 | A | GDP | eIF2gamma | Pyrococcus abyssi |
| 1jny | B | GDP | Elongation factor 1-alpha | Sulfolobus solfataricus |
| 3q5d | A | GDP | Atlastin-1 | Homo sapiens |
| 2r6r | 1 | GDP | Cell division protein ftsZ | Aquifex aeolicus VF5 |
| 2bm0 | A | GDP | ELONGATION FACTOR G | Thermus thermophilus |
| 2oxr | A | GDP | ATP(GTP)binding protein | Pyrococcus abyssi |
| 2xtm | A | GDP | GTPASE IMAP FAMILY MEMBER 2 | Homo sapiens |
| 2qth | A | GDP | GTP-binding protein | Sulfolobus solfataricus P2 |
| 1g7s | A | GDP | TRANSLATION INITIATION FACTOR IF2/EIF5B | Methanothermobacter thermautotrophicus |
| 2v40 | A | GDP | ADENYLOSUCCINATE SYNTHETASE ISOZYME 2 | Homo sapiens |
| 3fqj | A | GDP | Protein Dom3Z | Mus musculus |
| 1s4o | A | GDP | Glycolipid 2-alpha-mannosyltransferase | Saccharomyces cerevisiae |
| 2cxx | A | GDP | Probable GTP-binding protein engB | Pyrococcus horikoshii OT3 |
| 3ec1 | A | GDP | YqeH GTPase | Geobacillus stearothermophilus |
| 3e70 | C | GDP | Signal recognition particle receptor | Pyrococcus furiosus |
| 2h5e | A | GDP | Peptide chain release factor RF-3 | Escherichia coli |
| 2fpg | B | GDP | Succinyl-CoA ligase [GDP-forming] alpha-chain, mitochondrial | Sus scrofa |
| 3gj0 | B | GDP | GTP-binding nuclear protein Ran | Homo sapiens |
| 2zej | A | GDP | Leucine-rich repeat kinase 2 | Homo sapiens |
| 2wjh | A | GDP | FERROUS IRON TRANSPORT PROTEIN B HOMOLOG | Methanocaldococcus jannaschii |
| 2e87 | A | GDP | Hypothetical protein PH1320 | Pyrococcus horikoshii OT3 |
| 2yv5 | A | GDP | YjeQ protein | Aquifex aeolicus |
| 2ywg | A | GTP | GTP-binding protein LepA | Aquifex aeolicus VF5 |
| 1c80 | B | GTP | FRUCTOSE-2,6-BISPHOSPHATASE | Rattus norvegicus |
| 2r7w | A | GTP | RNA (5'-R(*UP*GP*UP*GP*AP*CP*C)-3') | Simian rotavirus |
| 1z0j | A | GTP | Ras-related protein Rab-22A | Mus musculus |
| 3moe | A | GTP | Phosphoenolpyruvate carboxykinase, cytosolic [GTP] | Rattus norvegicus |
| 1yr8 | A | GTP | ATP(GTP)binding protein | Pyrococcus abyssi |
| 2a8s | A | GTP | U8 snoRNA-binding protein X29 | Xenopus laevis |
| 3dco | A | GTP | Kinesin-like protein Nod | Drosophila melanogaster |
| 1ra7 | A | GTP | Genome polyprotein | Human poliovirus 1 Mahoney |
| 1p16 | B | GTP | mRNA capping enzyme alpha subunit | Candida albicans |
| 2jlg | C | GTP | RNA-DIRECTED RNA POLYMERASE | Pseudomonas phage phi6 |
| 2x60 | B | GTP | MANNOSE-1-PHOSPHATE GUANYLYLTRANSFERASE | Thermotoga maritima MSB8 |
| 3jqm | E | GTP | Molybdenum cofactor biosynthesis protein C | Thermus thermophilus HB8 |
| 1nrj | B | GTP | Signal recognition particle receptor alpha subunit homolog | Saccharomyces cerevisiae |
| 2irx | A | GTP | DNA ligase-like protein Rv0938/MT0965 | Mycobacterium tuberculosis H37Rv |
| 3agj | C | GTP | Elongation factor 1-alpha | Aeropyrum pernix |
| 1s49 | A | GTP | RNA-dependent RNA polymerase | Bovine viral diarrhea virus 1 |
| 3qxj | A | GTP | Dethiobiotin synthetase | Helicobacter pylori 26695 |
| 1c4k | A | GTP | PROTEIN (ORNITHINE DECARBOXYLASE) | Lactobacillus sp. 30A |
| 1w5b | B | GTP | CELL DIVISION PROTEIN FTSZ HOMOLOG 1 | Methanocaldococcus jannaschii |
| 2dy1 | A | GTP | Elongation factor G | Thermus thermophilus |
| 2fh5 | B | GTP | Signal recognition particle receptor alpha subunit | Homo sapiens |
| 2xtn | A | GTP | GTPASE IMAP FAMILY MEMBER 2 | Homo sapiens |
| 3ek5 | A | GTP | Uridylate kinase | Xanthomonas campestris pv. campestris |
| 3ldu | A | GTP | Putative methylase | Clostridium difficile 630 |
| 1loo | A | GTP | adenylosuccinate synthetase | Mus musculus |
| 2qv6 | A | GTP | GTP cyclohydrolase III | Methanocaldococcus jannaschii |
| 2xi3 | B | GTP | RNA-DIRECTED RNA POLYMERASE | Hepatitis C virus |
| 1ckm | B | GTP | MRNA CAPPING ENZYME | Paramecium bursaria Chlorella virus 1 |
| 3ffu | B | GTP | Probable pyrophosphohydrolase | Bdellovibrio bacteriovorus |
| 1a8r | A | GTP | GTP CYCLOHYDROLASE I | Escherichia coli |
| 2fp4 | B | GTP | Succinyl-CoA ligase [GDP-forming] alpha-chain, mitochondrial | Sus scrofa |
| 3dzh | A | GTP | ADP-ribosyl cyclase 1 | Homo sapiens |
| 2q0e | A | GTP | RNA uridylyl transferase | Trypanosoma brucei |
| 3m1i | A | GTP | GTP-binding nuclear protein GSP1/CNR1 | Saccharomyces cerevisiae |
| 2zgz | A | GNP | Plasmid segregation protein parM | Escherichia coli |
| 1day | A | GNP | PROTEIN KINASE CK2 | Zea mays |
| 1cip | A | GNP | PROTEIN (GUANINE NUCLEOTIDE-BINDING PROTEIN ALPHA-1 SUBUNIT) | Rattus norvegicus |
| 3ftq | C | GNP | Septin-2 | Mus musculus |
| 3bb4 | A | GNP | T7I23.11 protein | Arabidopsis thaliana |
| 1wf3 | A | GNP | GTP-binding protein | Thermus thermophilus HB8 |
| 1r2q | A | GNP | Ras-related protein Rab-5A | Homo sapiens |
| 2ywf | A | GNP | GTP-binding protein lepA | Aquifex aeolicus VF5 |
| 3a1u | B | GNP | Iron(II) transport protein B | Thermotoga maritima |
| 2j7p | E | GNP | SIGNAL RECOGNITION PARTICLE PROTEIN | Thermus aquaticus |
| 1tq2 | A | GNP | interferon-inducible GTPase | Mus musculus |
| 1puj | A | GNP | conserved hypothetical protein ylqF | Bacillus subtilis |
| 2px0 | B | GNP | Flagellar biosynthesis protein flhF | Bacillus subtilis |
| 1kk1 | A | GNP | eIF2gamma | Pyrococcus abyssi |
| 2c77 | A | GNP | ELONGATION FACTOR TU-B | Thermus thermophilus HB8 |
| 2bv3 | A | GNP | ELONGATION FACTOR G | Thermus thermophilus |
| 1sz3 | A | GNP | MutT/nudix family protein | Deinococcus radiodurans |
| 1g7t | A | GNP | TRANSLATION INITIATION FACTOR IF2/EIF5B | Methanothermobacter thermautotrophicus |
| 3llu | A | GNP | Ras-related GTP-binding protein C | Homo sapiens |
| 1f5n | A | GNP | INTERFERON-INDUCED GUANYLATE-BINDING PROTEIN 1 | Homo sapiens |
| 2wji | A | GNP | FERROUS IRON TRANSPORT PROTEIN B HOMOLOG | Methanocaldococcus jannaschii |
| 1ibr | A | GNP | GTP-binding nuclear protein RAN | Homo sapiens |

**Table S1**. List of the 924 protein structures included in the sc-PDB dataset. The table reports the PDB code, the chain of the protein structure analyzed by the method, the nucleotide bound by the protein, the protein name and name of the organism. Proteins are grouped by the type of nucleotide bound.
