# Supplementary material for: Identification of Nucleotide-Binding Sites in Protein Structures: A Novel Approach Based on Nucleotide Modularity
Source: PLoS One. 2012 Nov 27;7(11):e50240. doi: 10.1371/journal.pone.0050240 (PMC3507729; doi:10.1371/journal.pone.0050240)
Supplement: Table S2 — List of the 64 protein structures included in the LigASite dataset. The table reports the PDB code of the apo and holo structures (grouped by the type of nucleotide bound) of the same protein, the chain of the protein structures analyzed by the method, the nucleotide bound by the protein, the protein name and name of the organism. (DOCX) [file pone.0050240.s002.docx]

| **Apo**  **PDB** | **Chain** | **Holo**  **PDB** | **Chain** | **Ligand** | **Protein name** | **Species** |
| --- | --- | --- | --- | --- | --- | --- |
| 1o24 | D | 1o26 | D | FAD | Thymidylate synthase thyX | Thermotoga maritima |
| 1ws9 | A | 2d29 | A | FAD | Acyl-CoA dehydrogenase | Thermus thermophilus HB8 |
| 2oam | A | 2oa1 | A | FAD | Tryptophan halogenase | Lechevalieria aerocolonigenes |
| 1b8p | A | 1b8u | A | NAD | Malate Dehydrogenase | Aquaspirillum arcticum |
| 1crw | G | 1szj | G | NAD | D-Glyceraldehyde-3-Phosphate-Dehydrogenase | Panulirus versicolor |
| 1ojq | A | 1ojz | A | NAD | ADP-Ribosyltransferase | Staphylococcus aureus |
| 1wxf | A | 1wxh | A | NAD | NH(3)-dependent NAD(+) synthetase | Escherichia coli |
| 2wn4 | A | 2wn7 | A | NAD | ADP-Ribosyltransferase Enzymatic Component | Clostridium difficile |
| 3btv | B | 3bts | B | NAD | Galactose/lactose metabolism regulatory protein GAL80 | Saccharomyces cerevisiae |
| 3d0o | B | 3d4p | B | NAD | L-lactate dehydrogenase 1 | Staphylococcus aureus subsp. aureus COL |
| 3etf | C | 3efv | C | NAD | Putative succinate-semialdehyde dehydrogenase | Salmonella enterica subsp. enterica serovar Typhimurium |
| 1gfs | A | 1fxs | A | NAP | GDP-Fucose Synthetase | Escherichia coli K-12 |
| 1ulu | D | 2yw9 | D | NAP | Enoyl-[acyl carrier protein] reductase | Thermus thermophilus HB8 |
| 2e0c | B | 2e5m | B | NAP | 409aa long hypothetical NADP-dependent isocitrate dehydrogenase | Sulfolobus tokodaii str. 7 |
| 2gqv | A | 2rk2 | A | NAP | Dihydrofolate reductase type 2 | Escherichia coli |
| 2gyy | A | 2gz3 | A | NAP | Aspartate beta-semialdehyde dehydrogenase | Streptococcus pneumoniae |
| 3ba1 | A | 3baz | A | NAP | Hydroxyphenylpyruvate reductase | Solenostemon scutellarioides |
| 1umf | A | 1um0 | A | FMN | Chorismate synthase | Helicobacter pylori |
| 1w9a | A | 1y30 | A | FMN | hypothetical protein Rv1155 | Mycobacterium tuberculosis |
| 2jbr | A | 2jbt | A | FMN | P-Hydroxyphenylacetate Hydroxylase C2\:Oxygenase Component | Acinetobacter baumannii |
| 1m1z | A | 1mc1 | A | AMP | Beta-Lactam Synthetase | Streptomyces clavuligerus |
| 2hbj | A | 2hbl | A | AMP | Exosome complex exonuclease RRP6 | Saccharomyces cerevisiae |
| 1mwk | A | 2zhc | A | ADP | Plasmid segregation protein parM | Escherichia coli |
| 1rzv | B | 1rzu | B | ADP | Glycogen synthase 1 | Agrobacterium tumefaciens |
| 1vfj | C | 1v9o | C | ADP | Nitrogen Regulatory Protein PII | Thermus thermophilus |
| 2c61 | A | 3dsr | A | ADP | V-type ATP synthase beta chain | Methanosarcina mazei |
| 2cwk | A | 2dya | A | ADP | Nucleoside diphosphate kinase | Pyrococcus horikoshii OT3 |
| 2q5r | D | 2jgv | D | ADP | Tagatose-6-Phosphate Kinase | Staphylococcus aureus subsp. aureus NCTC 8325 |
| 2yzg | C | 2zdg | C | ADP | D-alanine--D-alanine ligase | Thermus thermophilus HB8 |
| 2zj8 | A | 2zj5 | A | ADP | Putative ski2-type helicase | Pyrococcus furiosus |
| 3a0y | A | 3a0t | A | ADP | Sensor protein | Thermotoga maritima |
| 3dre | B | 3drb | B | ADP | Creatine kinase B-type | Homo sapiens |
| 3fv6 | A | 3fwr | A | ADP | YqzB protein | Bacillus subtilis |
| 3h49 | B | 3in1 | B | ADP | Uncharacterized sugar kinase ydjH | Escherichia coli K-12 |
| 3kaj | A | 3kal | A | ADP | homoglutathione synthetase | Glycine max |
| 3kje | A | 3kji | A | ADP | CO dehydrogenase/acetyl-CoA synthase complex, accessory protein CooC | Carboxydothermus hydrogenoformans Z-2901 |
| 4ake | B | 2eck | B | ADP | Adenylate Kinase | Escherichia coli |
| 1e4f | T | 1e4g | T | ATP | Cell Division Protein FTSA | Thermotoga maritima |
| 1hka | A | 1dy3 | A | ATP | 6-hydroxymethyl-7,8-dihydropterin pyrophosphokinase | Escherichia coli |
| 1i7n | B | 1i7l | B | ATP | Synapsin II | Rattus norvegicus |
| 1riq | A | 1yfr | A | ATP | Alanyl-tRNA synthetase | Aquifex aeolicus |
| 1sjy | A | 1su2 | A | ATP | MutT/nudix family protein | Deinococcus radiodurans |
| 1yvy | A | 1ytm | A | ATP | Phosphoenolpyruvate carboxykinase | Anaerobiospirillum succiniciproducens |
| 2bjw | A | 2c96 | A | ATP | PSP Operon Transcriptional Activator | Escherichia coli K-12 |
| 2c7i | A | 2aru | A | ATP | Lipoate-protein ligase A | Thermoplasma acidophilum |
| 2fsf | A | 2fsg | A | ATP | Preprotein translocase secA subunit | Escherichia coli |
| 2hiv | A | 2hix | A | ATP | Thermostable DNA ligase | Sulfolobus solfataricus |
| 2i4l | B | 2i4o | B | ATP | Proline-tRNA ligase | Rhodopseudomonas palustris |
| 2yya | A | 2yw2 | A | ATP | Phosphoribosylamine--glycine ligase | Aquifex aeolicus |
| 2zhy | C | 2zhz | C | ATP | ATP:cob(I)alamin adenosyltransferase, putative | Burkholderia thailandensis |
| 3gbt | A | 3ll3 | A | ATP | Gluconate kinase | Lactobacillus acidophilus |
| 3gqh | A | 3gqk | A | ATP | Preneck appendage protein | Bacillus phage phi29 |
| 3h38 | A | 3h39 | A | ATP | TRNA nucleotidyl transferase-related protein | Thermotoga maritima |
| 1jcf | A | 1jcg | A | ANP | Rod Shape-Determining Protein Mreb | Thermotoga maritima |
| 1jks | A | 1jkl | A | ANP | Death-Associated Protein Kinase | Homo sapiens |
| 2gsf | A | 2qo9 | A | ANP | Ephrin receptor | Homo sapiens |
| 2v78 | A | 2var | A | ANP | Fructokinase | Sulfolobus solfataricus |
| 3a2r | X | 3a2u | X | ANP | Outer membrane protein II | Neisseria meningitidis |
| 3aap | A | 3aar | A | ANP | Ectonucleoside triphosphate diphosphohydrolase I | Legionella pneumophila subsp. pneumophila str. Philadelphia 1 |
| 1znw | A | 1znz | A | GDP | Guanylate kinase | Mycobacterium tuberculosis |
| 3i8s | A | 3i8x | A | GDP | Ferrous iron transport protein B | Escherichia coli |
| 1sul | B | 1svw | B | GTP | GTP-binding protein YsxC | Bacillus subtilis |
| 2x5s | B | 2x60 | B | GTP | Mannose-1-phosphate guanylyltransferase | Thermotoga maritima MSB8 |
| 3ek6 | A | 3ek5 | A | GTP | Uridylate kinase | Xanthomonas campestris pv. campestris |

**Table S2**. List of the 64 protein structures included in the LigASite dataset. The table reports the PDB code of the apo and holo structures (grouped by the type of nucleotide bound) of the same protein, the chain of the protein structures analyzed by the method, the nucleotide bound by the protein, the protein name and name of the organism.
